# Nearly-incompressible transverse isotropy (NITI) of cornea elasticity: model and experiments with acoustic micro-tapping OCE

**DOI:** 10.1038/s41598-020-69909-9

**Published:** 2020-07-31

**Authors:** John J. Pitre, Mitchell A. Kirby, David S. Li, Tueng T. Shen, Ruikang K. Wang, Matthew O’Donnell, Ivan Pelivanov

**Affiliations:** 1 Department of Bioengineering, University of Washington, Seattle, WA USA; 2 Department of Chemical Engineering, University of Washington, Seattle, WA USA; 3 Department of Ophthalmology, University of Washington, Seattle, WA USA

**Keywords:** Imaging and sensing, Biological physics, Corneal diseases, Biomedical engineering, Medical imaging

## Abstract

The cornea provides the largest refractive power for the human visual system. Its stiffness, along with intraocular pressure (IOP), are linked to several pathologies, including keratoconus and glaucoma. Although mechanical tests can quantify corneal elasticity ex vivo, they cannot be used clinically. Dynamic optical coherence elastography (OCE), which launches and tracks shear waves to estimate stiffness, provides an attractive non-contact probe of corneal elasticity. To date, however, OCE studies report corneal moduli around tens of kPa, orders-of-magnitude less than those (few MPa) obtained by tensile/inflation testing. This large discrepancy impedes OCE’s clinical adoption. Based on corneal microstructure, we introduce and fully characterize a nearly-incompressible transversely isotropic (NITI) model depicting corneal biomechanics. We show that the cornea must be described by at least two shear moduli, contrary to current single-modulus models, decoupling tensile and shear responses. We measure both as a function of IOP in ex vivo porcine cornea, obtaining values consistent with both tensile and shear tests. At pressures above 30 mmHg, the model begins to fail, consistent with non-linear changes in cornea at high IOP.

## Introduction

**Figure 1:**
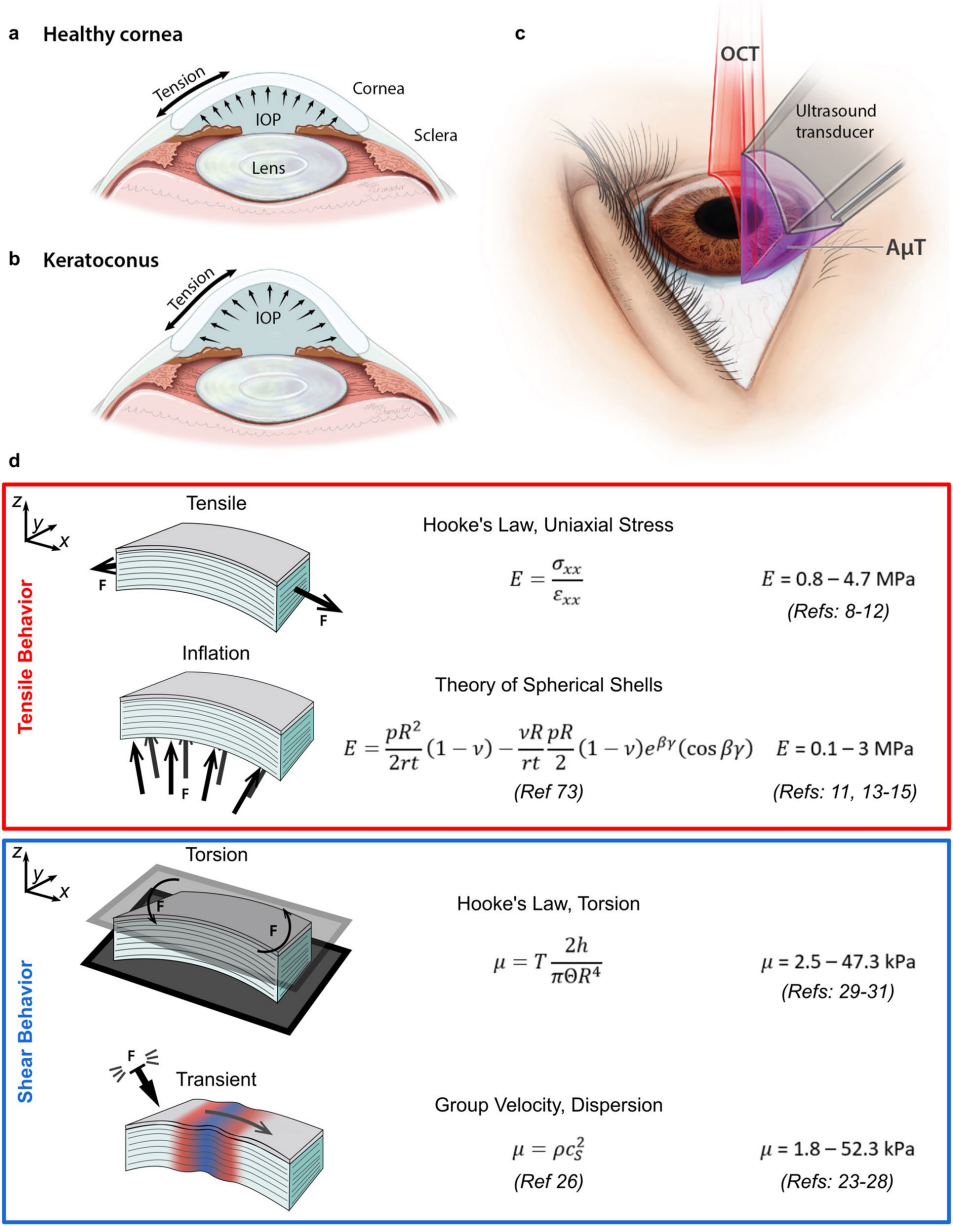
Relationships between mechanical forces in the cornea and various mechanical testing methods. Mechanical forces in corneal tissue govern focusing power in (**a**) healthy and (**b**) pathological tissue (e.g. keratoconus). (**c**) Elastic shear waves generated in the cornea with a transient force are tracked using optical coherence tomography to determine corneal mechanical properties without contact. (**d**) Summary of biomechanical test methods and corneal Young’s and shear moduli from the literature. Reported moduli vary by up to four orders of magnitude depending on the loading technique and test configuration, suggesting that isotropic models cannot accurately describe corneal mechanics. In particular, tensile and inflation tests generally agree, as do shear and transient tests. Values listed are for fresh samples (within 2 weeks), and best effort was taken to report moduli in the low-strain/preload region where they generally are near their lowest value.

The human cornea provides a unique combination of structure and function to the visual system, serving as a transparent barrier providing two-thirds of the eye’s refractive power^1^. It forms a clear refractive lens because of its structure, consisting of collagen fibrils embedded in a hydrated proteoglycan matrix, and is the primary determinant of visual performance^2^. Indeed, its constituents’ mechanical properties regulate shape (Fig. 1a), helping focus light onto the retina^3,4^.

Because corneal function depends on stiffness, biomechanical metrics can be valuable to both understand and treat corneal disease. For example, keratoconic corneas (Fig. 1b) are measurably less stiff than healthy ones^5^. This observation led to clinical interventions increasing stiffness (e.g. cornea cross-linking)^6^. In procedures such as LASIK and photorefractive keratectomy (PRK), an incision releases stromal tension, inducing structural changes that adjust focusing^4^. This close relationship between mechanics and function defines a clinical need for simple and robust measures of corneal stiffness.

In practice, tissue microstructure is too complex to model directly, so assumptions are made to simplify mechanical descriptions of the cornea. The most common model assumes it is a nearly incompressible, isotropic, and linear elastic solid. For this case, a single elastic parameter, the Young’s modulus *E* (or equivalently the shear modulus μ = *E*/3), defines stiffness. It has been correlated with a number of pathologies and used to design interventions^7^. Unfortunately, measurements require ex vivo tissue samples loaded under tension or inflation. These destructive methods accurately determine corneal *E*, with reported values for human cornea (in the low-strain region) of 800 kPa to 4.7 MPa for tensile loading^8–12^, and 100 kPa to 3 MPa for inflation loading^11,13–15^. Although they provide important information on corneal mechanics, their destructive nature precludes clinical translation. Thus, there is clear need for a reliable, non-contact, and non-invasive method to measure corneal biomechanical properties in vivo*.*

Optical coherence elastography (OCE) is a promising tool to probe corneal biomechanics. OCE encompasses multiple methods combining an applied mechanical compression^16–18^, harmonic^19–21^, or transient^22–25^ excitation with high-resolution optical coherence tomography imaging to estimate elastic properties. Dynamic OCE in particular may be able to deliver a non-contact and non-invasive measurement in a clinical environment^26,27^ as it excites elastic shear waves in the cornea (for example, using an air-puff or acoustic micro-tapping, AμT^22^) and tracks them using optical coherence tomography (OCT) (Fig. 1c). Analyzing the shear wave group velocity or dispersion leads to an estimate of tissue shear modulus μ. OCE studies have reported corneal shear moduli in the range of 1.8–52.3 kPa^23,28^, in close agreement with values obtained from torsional testing of ex vivo cornea (2.5–47.3 kPa)^29–31^. However, both shear-based methods produce moduli differing by 1–2 orders of magnitude from those reported by tensile and inflation tests (assuming isotropy, *E* = 3μ) (Fig. 1d).

Clearly, cornea exhibits markedly different stiffness under shear versus tensile loading, indicating that it cannot be fully described by the single material parameter *E*. Still, the vast majority of corneal mechanics studies over the past 50 years have reported a single Young’s modulus, leading to multiple order-of-magnitude discrepancies between results from the two classes of mechanical tests (tensile and shear). Supplementary Note 1 presents a more complete summary of reported Young’s and shear moduli, and how they were measured.

We hypothesize that anisotropy is the primary cause of discrepancies between tensile/inflation and torsional/OCE modulus measurements. Corneal microstructure supports this hypothesis. The stroma contains collagen lamellae running in-plane across its width. They make up approximately 90% of tissue thickness and account for the majority of the cornea’s mechanical structure. Lamellae are stacked vertically in approximately 200–500 separate planes, with various levels of complexity along depth^32,33^, resulting in anisotropic structure and behavior. High-resolution imaging, combined with reported moduli for different loading schemes, strongly suggest that the cornea is anisotropic.

Our goal is to develop an OCE-based technique for clinically translatable measurements consistent with direct mechanical estimates. To obtain reliable, quantitative measurements of corneal moduli, we must address multiple aspects of mechanical wave propagation considering corneal structure. The cornea’s finite thickness, bounded by air on one side and liquid on the other, produces complicated guided wave behavior^26^. Adding tissue anisotropy complicates this further, and estimation of moduli is even more difficult. Solutions are not trivial, especially when tissue anisotropy between tensile and shear loading regimes must be considered.

Here we propose a transversely isotropic (TI) model of the cornea decoupling shear from tensile behavior, thus resolving the apparent paradox in reported biomechanical properties. It intuitively links cornea microstructure to the observed mechanical response, where collagen lamellae contribute to the stiff behavior under tension and inflation (MPa range), while the layered structure allows internal slip producing the softer response of shear and transient tests (kPa range). We compare analytical and simulation results to OCE measurements and demonstrate that the TI model greatly improves quantitative estimates of corneal moduli that agree with ex vivo mechanical tests. These results suggest that clinical stiffness measurements made with OCE may be used to connect the field of non-destructive in vivo testing with the extensive body of ex vivo literature. Bridging this gap is an important step in clinically translating OCE and may help launch large clinical studies in the future.

## Results

### Nearly incompressible transversely isotropic (NITI) model of cornea

Corneal stroma contains hundreds of vertically stacked collagen lamellae, each 0.2–2.5 μm thick^34^ with a preferred collagen orientation. While some in-plane anisotropy has been reported^28,35–38^, its magnitude at low intraocular pressure (IOP) suggests that macroscopic behavior can be treated as isotropic in-plane^22,38,39^. Recent second harmonic generation imaging studies show that lamellar orientations are more random than previously suggested^32,40,41^, further supporting the assumption of in-plane isotropy. One interpretation of this structure, based on a fiber-composite model^42^, is that collagen fiber mechanical properties govern in-plane behavior, while those of the connective tissue matrix govern out-of-plane behavior.

A transversely isotropic (TI) model is the most appropriate given an isotropy plane. It contains five independent elastic constants (*C*_11_, *C*_12_, *C*_13_, *C*_33_, *C*_44_) rather than the two (Lamé constants) of isotropic materials. To simplify notation, we adopt the shorthand: *C*_11_ = λ + 2μ, *C*_12_ = λ, and *C*_44_ = *G*. We also assume that the cornea, like most soft tissue, is nearly-incompressible. That is, the medium’s internal pressure remains finite as λ → ∞ and the dilatation approaches zero. When applied to a transversely isotropic solid, this assumption leads to a set of conditions on the longitudinal terms of the stiffness matrix (*C*_11_, *C*_12_, *C*_13_, *C*_33_). These conditions do not reduce the number of independent constants, but they do provide limiting relationships that define the nearly-incompressible condition^43^.

Of note, any transversely isotropic solid for which *C*_13_ and *C*_33_ are asymptotically equal to λ will behave as a nearly-incompressible solid, provided the stiffness matrix remains invertible (Supplementary Note 2). This last condition must be treated carefully. When the longitudinal part of the stiffness matrix is isotropic (*C*_13_ = *C*_12_ and *C*_11_ = *C*_22_ = *C*_33_), the stiffness matrix is always invertible. This condition corresponds to weak anisotropy, an assumption supported by Brillouin microscopy measurements^44^. We note that more exact estimates of *C*_13_ and *C*_33_ can be independently measured with Brillouin microscopy; however, these terms contribute little to predicting mechanical (shear, surface, guided) wave behavior in nearly-incompressible solids, i.e. have very little effect on shear moduli inversion from dynamic elastography measurements (Supplementary Note 4).

Thus, we take *C*_13_ = λ and *C*_33_ = λ + 2μ and finally define the stiffness tensor of a nearly-incompressible transversely isotropic (NITI) material as:

$$C=\left[\begin{array}{cccccc}\lambda +2\mu & \lambda & \lambda & & & \\ \lambda & \lambda +2\mu & \lambda & & & \\ \lambda & \lambda & \lambda +2\mu & & & \\ & & & G& & \\ & & & & G& \\ & & & & & \mu \end{array}\right].$$

The constants λ and μ mimic those in an isotropic solid, with λ enforcing incompressibility and μ defining in-plane shear, tensile, and compressive behavior. Similar to an isotropic material, the Young’s modulus is simply related to μ, *E* = 3μ. An additional shear constant *G* governs out-of-plane shear and is completely decoupled from *E*.

Uniaxial tensile and inflation tests yield Young’s modulus estimates related only to μ. However, shear torsional tests depend only on *G*. In addition, the speed of vertically-polarized bulk shear waves is a function of *G*. This decoupling of normal and shear deformations helps explain the discrepancy between tensile/inflation test modulus estimates (on the order of MPa) and shear/transient estimates (on the order of kPa). Supplementary Note 2 shows how to obtain NITI parameters μ and *G* from tensile, inflation, shear, and transient mechanical tests.

### Wave behavior in a bulk NITI medium

Unlike isotropic materials, which support only two bulk waves (longitudinal and shear), transversely isotropic materials support three—quasi-longitudinal, quasi-shear, and shear. Soft tissue is nearly-incompressible (λ ≫ μ), implying that the quasi-longitudinal wave speed is nearly constant over all directions of a NITI medium. The anisotropy of longitudinal wave propagation in cornea has been recently shown to be very weak, with angular variations in the quasi-longitudinal wave speed < 5%^44^. In contrast, quasi-shear and shear wave speeds vary dramatically with angle and depend on both *G* and μ. This has important implications for OCE measurements.

Many dynamic OCE methods track mechanical waves propagating along the air-cornea interface, ignoring liquid loading on the cornea’s posterior surface^28,36,45,46^. In other words, the cornea is considered semi-infinite with a simple Rayleigh wave propagating along the surface. The in-plane Rayleigh wave speed can be obtained numerically from the Stroh formalism^47–51^. For materials with *G* < μ, such as we expect for cornea, the Rayleigh wave speed varies from *c*_*R*_ = $\sqrt{G/\rho }$ in the highly anisotropic limit (*G* ≪  μ) to *c*_*R*_ = 0.9553 $\sqrt{G/\rho }$ in the isotropic limit (*G* = μ) (Supplementary Note 3^,^Fig. S3.2). Even for varying degrees of anisotropy, Rayleigh wave speed is primarily governed by *G* and only slightly influenced by μ. This leads to an important conclusion. If the cornea could be considered a semi-infinite NITI medium, it would be extremely difficult to determine μ from OCE measurements. For reference, Supplementary Note 3 derives bulk and Rayleigh wave speeds in a NITI medium.

In reality, the cornea’s thickness is typically on the order of the wavelength of propagating mechanical waves considered in dynamic OCE^22,26,52^. As such, corneal thickness and boundary conditions lead to guided waves. As shown below, guided waves provide additional information to help extract both *G* and μ from dynamic OCE measurements.

### Guided wave behavior in a bounded NITI layer

**Figure 2:**
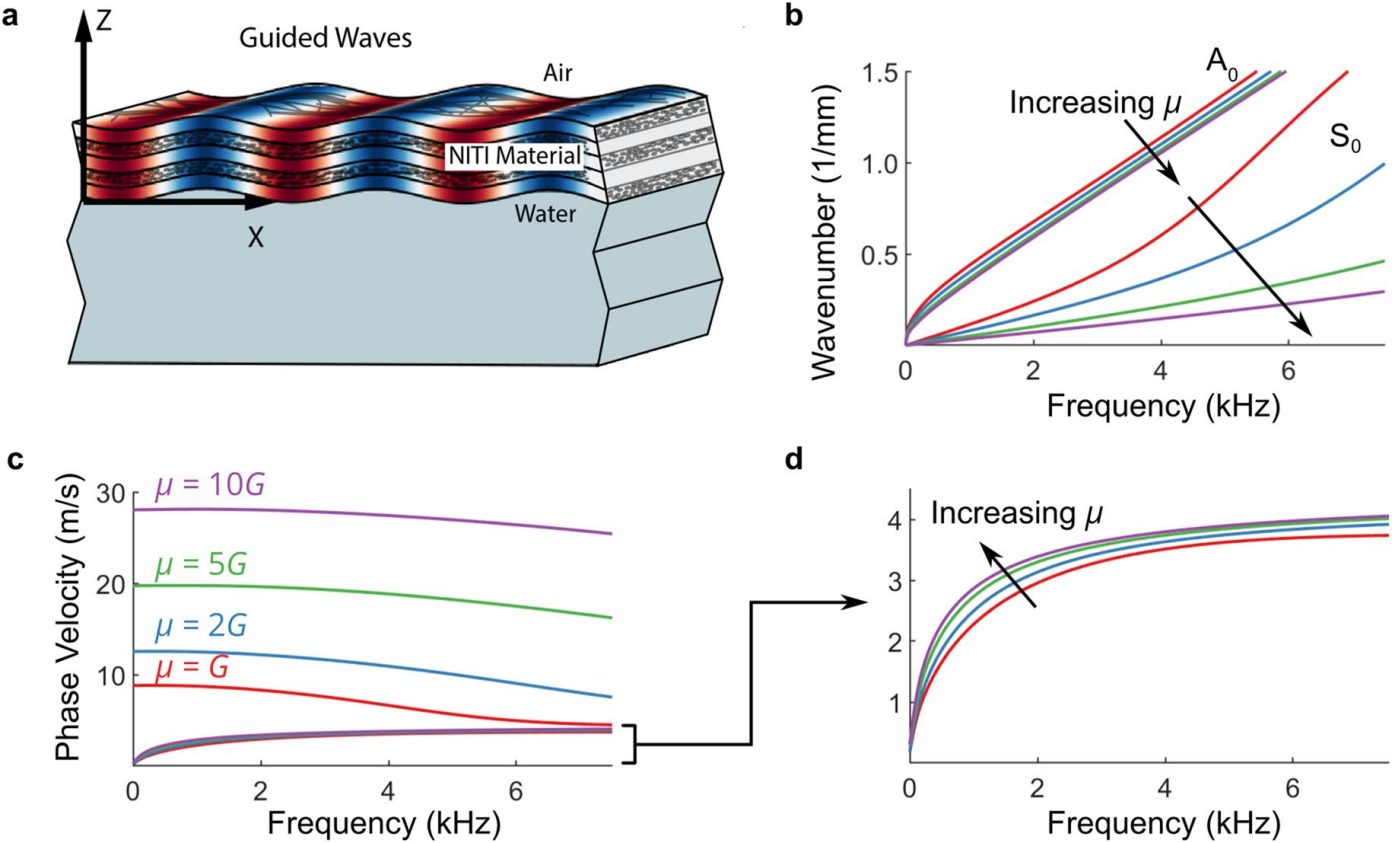
Analytical solutions for guided wave behavior in a bounded NITI layer. (**a**) Propagation of harmonic guided waves in cornea tissue, modeled as a NITI material layer bounded above by air and below by water. Dispersion relations for propagating guided modes are derived from partial wave solutions to the elastic wave equations satisfying corneal boundary conditions in Supplementary Note 4. (**b**) The effect of increasing anisotropy on fundamental (zero order) A_0_ and S_0_ modes (*G* = 20 kPa, *h* = 0.55 mm, varying μ) presented in wavenumber-frequency space. The behavior of the A_0_ mode is primarily governed by *G*, and the S_0_ mode by μ. (**c**) The same dispersion curves in phase velocity versus frequency representation. (**d**) Zoomed view of the A_0_ mode in phase velocity versus frequency representation—the high-frequency asymptote of the A_0_ mode depends primarily on *G*, with its low-frequency rate of change governed by μ.

The cornea’s bounded geometry produces dispersive guided waves, which must be analyzed in frequency-wavenumber (ω-k) space to quantify elasticity. Partial wave analysis assuming the cornea as a flat isotropic layer bounded by air and water (Fig. 2a) leads to a secular equation describing guided modes^23,26^. Here, we introduce the dispersion relation for a NITI layer bounded by air and water, derived from partial wave solutions to the elastic wave equations satisfying corneal boundary conditions. Supplementary Note 4 provides a full derivation, and functions solving this dispersion relation are provided in Supplementary Software.

While the solution includes an infinite number of quasi-antisymmetric (A) and quasi-symmetric modes (S), only the first two (A_0_ and S_0_) are typically captured in OCE. Figure 2 shows some examples for NITI materials with varying levels of anisotropy (*G* = 20 kPa, *h* = 0.55 mm, varying μ). As μ increases, the S_0_ mode propagates with higher phase velocity at low frequencies (Fig. 2c). In contrast, the high frequency asymptote of the A_0_ mode is primarily governed by *G*, with μ controlling both the rate at which the A_0_ mode approaches its asymptote and weakly affecting the asymptotic value (< 5%) (Fig. 2d). Thus, the cornea’s bounded structure provides a potential avenue to determine both *G* and μ using OCE.

### Finite element model of guided waves in a bounded NITI medium

Although an analytical solution exists, it is not valid near the mechanical wave excitation source (near field) and does not describe how energy is distributed among guided modes in a NITI layer. To address this, we developed a finite element model using OnScale (OnScale, Redwood City, CA). It contains a NITI layer bounded above by air and below by water with dimensions similar to the cornea. Elastic waves were excited with a spatio-temporally short pressure applied to the top surface, mimicking AμT. Supplementary Note 5 presents a detailed model description, and the OnScale input file is available in Supplementary Software.

**Figure 3:**
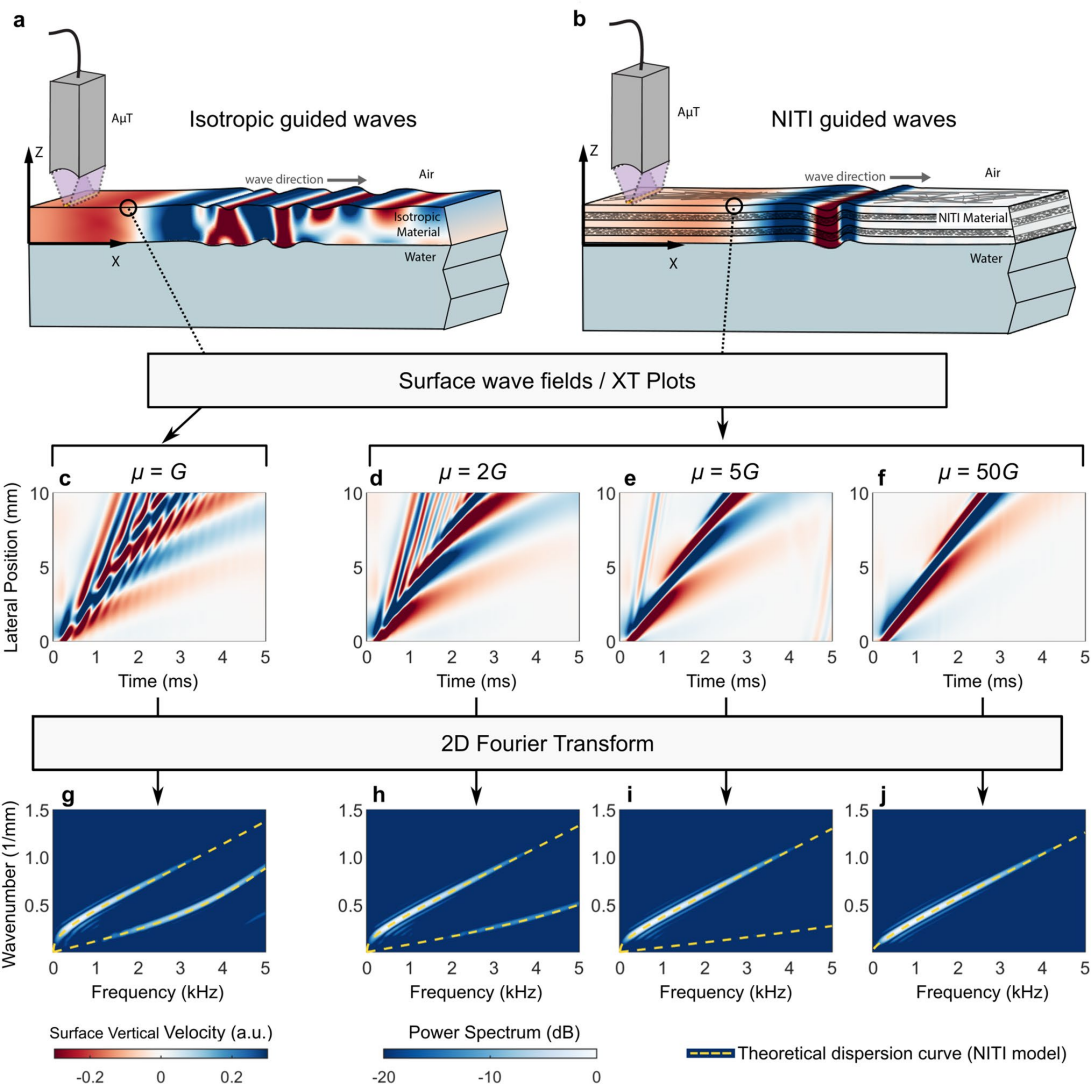
Numerical solutions for guided wave propagation in a bounded NITI medium. Guided mode wave fields simulated in OnScale for (**a**) isotropic and (**b**) NITI layers. Guided wave excitation was simulated with the AμT line source, closely mimicking experimental conditions (see Supplementary Note 5). Extracting guided mode wave fields from the material surface yields XT plots (**c**–**f**) and corresponding 2D Fourier spectra (**g**–**j**, presented on a log scale over a 20 dB display dynamic range) for various levels of anisotropy. A 0.55 mm thick NITI layer (*G* = 20 kPa; μ = 20 kPa, 40 kPa, 100 kPa, 1 MPa) is considered.

We examined temporal changes in the surface velocity field over a range of lateral positions. Unitless surface vibrations illustrate the spatio-temporal behavior of the surface wave field and mimic an OCE measurement (Fig. 3a,b). This is referred to as an XT plot. A two-dimensional Fourier transform was then applied to analyze guided wave dispersion. Figure 3 shows XT plots and corresponding 2D Fourier spectra for increasing μ. In the isotropic limit (μ = *G*), the XT plot (Fig. 3c) clearly shows multiple interacting guided waves, with A_0_ and S_0_ modes both visible in the spectrum (Fig. 3g). As anisotropy increases (μ > *G*), XT plots (Fig. 3d–f) change dramatically. Interference between A_0_ and S_0_ modes decreases at μ = 2*G* and is nearly absent at μ = 5*G*. Furthermore, as μ increases, the shapes of A_0_ and S_0_ modes shift. Energy in the S_0_ mode also decreases, disappearing almost entirely for μ ≥ 5*G* (Fig. 3i,j). These results strongly suggest that if the NITI model is valid, then only one mode, A_0_, should be visible in cornea. This is in contrast to an isotropic layer of the same thickness, where multiple high order modes can be observed.

### Elastic modulus estimates with AμT-driven OCE

A spectral-domain OCT system with a 46.5 kHz effective frame rate, as detailed previously^53^, tracked guided waves in isotropic polyvinyl alcohol (PVA) cryogels and porcine cornea, providing experimental measurements to compare with theoretical predictions. A cylindrically-focused 1 MHz air-coupled ultrasound transducer (AμT) provided a spatio-temporally sharp push to the surface of a thin isotropic PVA phantom or freshly excised porcine cornea (n = 6) with IOP incrementally increasing from 5 to 35 mmHg, generating mechanical waves with bandwidths up to 4 kHz (“Methods” section).

**Figure 4:**
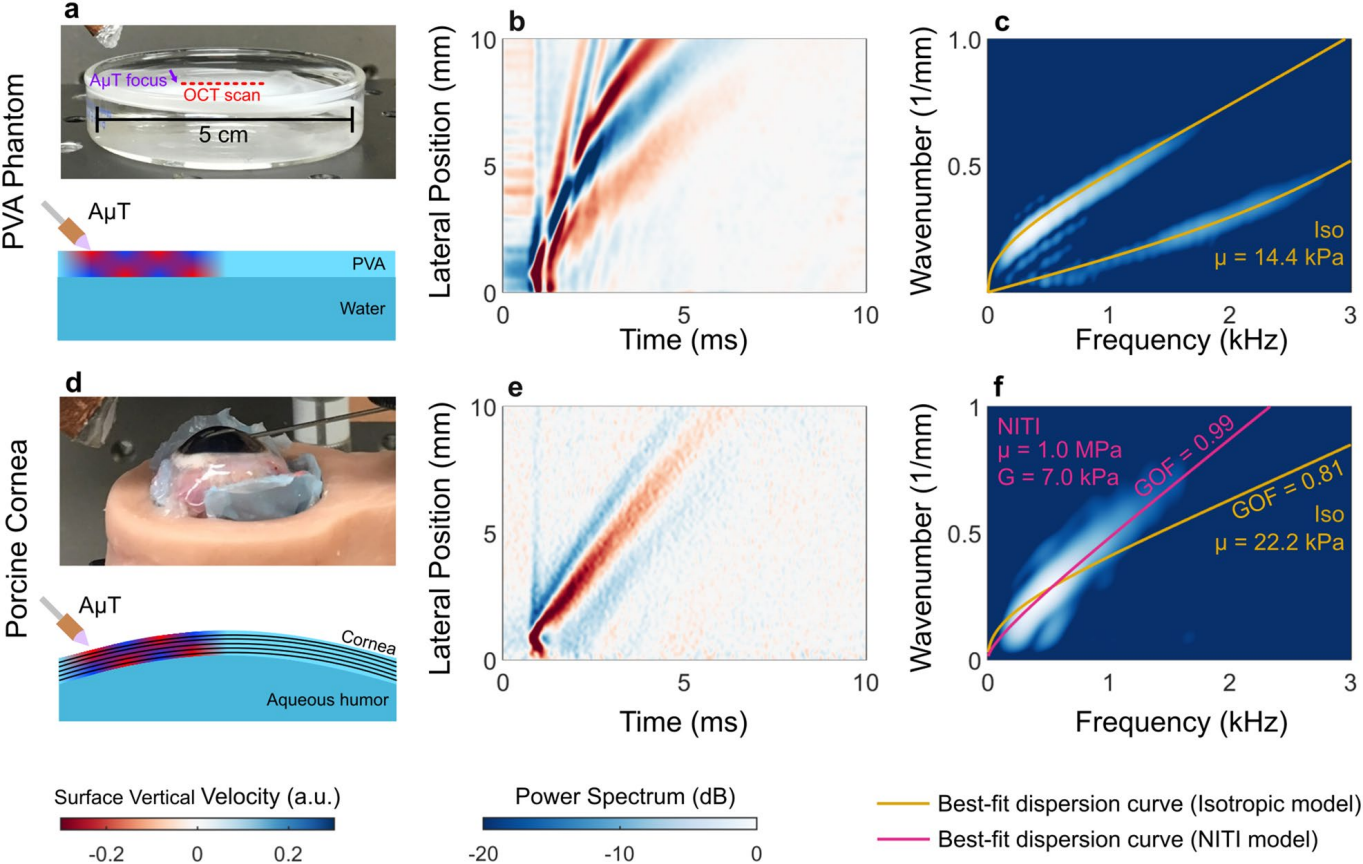
AμT-OCE estimation of elastic moduli in an isotropic phantom and ex vivo porcine cornea. (**a**) OCE experiments in an isotropic thin PVA phantom bounded above by air and below by water. (**b**) The guided wave fields excited by AμT are extracted at the phantom surface (XT plot) and a 2D Fourier transform is applied to obtain the frequency-wavenumber spectrum (**c**, presented on a log scale over a 20 dB display dynamic range). Dispersion curves for an isotropic material are then fit to the spectrum (**c**, yellow line). The behavior is markedly different from porcine cornea (**d**) in both the XT plot (**e**) and wavenumber-frequency spectrum (**f**). Isotropic and NITI model best fits are shown in (**f**) along with parameter estimates and goodness-of-fit metrics (GOF, see Supplementary Note 7).

Figure 4 compares OCE-measured surface velocity fields obtained for an isotropic PVA phantom (Fig. 4a) and porcine cornea at an IOP of 5 mmHg (Fig. 4d). Guided waves are apparent in the isotropic phantom (Fig. 4b). The 2D spectrum of this XT wave field clearly has two guided modes, and fitting an isotropic dispersion relation^26^ yields a shear modulus estimate, μ_*PVA*_ = 14.4 kPa (Fig. 4c, yellow curves).

Porcine cornea displays very different behavior, with the wave energy concentrated in a single dispersive mode (Fig. 4e). Two-dimensional spectra highlight differences between PVA and cornea wave fields (Fig. 4c,f). Clearly, only the A_0_ mode is present in porcine cornea (Fig. 4f), as predicted by numerical simulations (Fig. 3).

**Figure 5:**
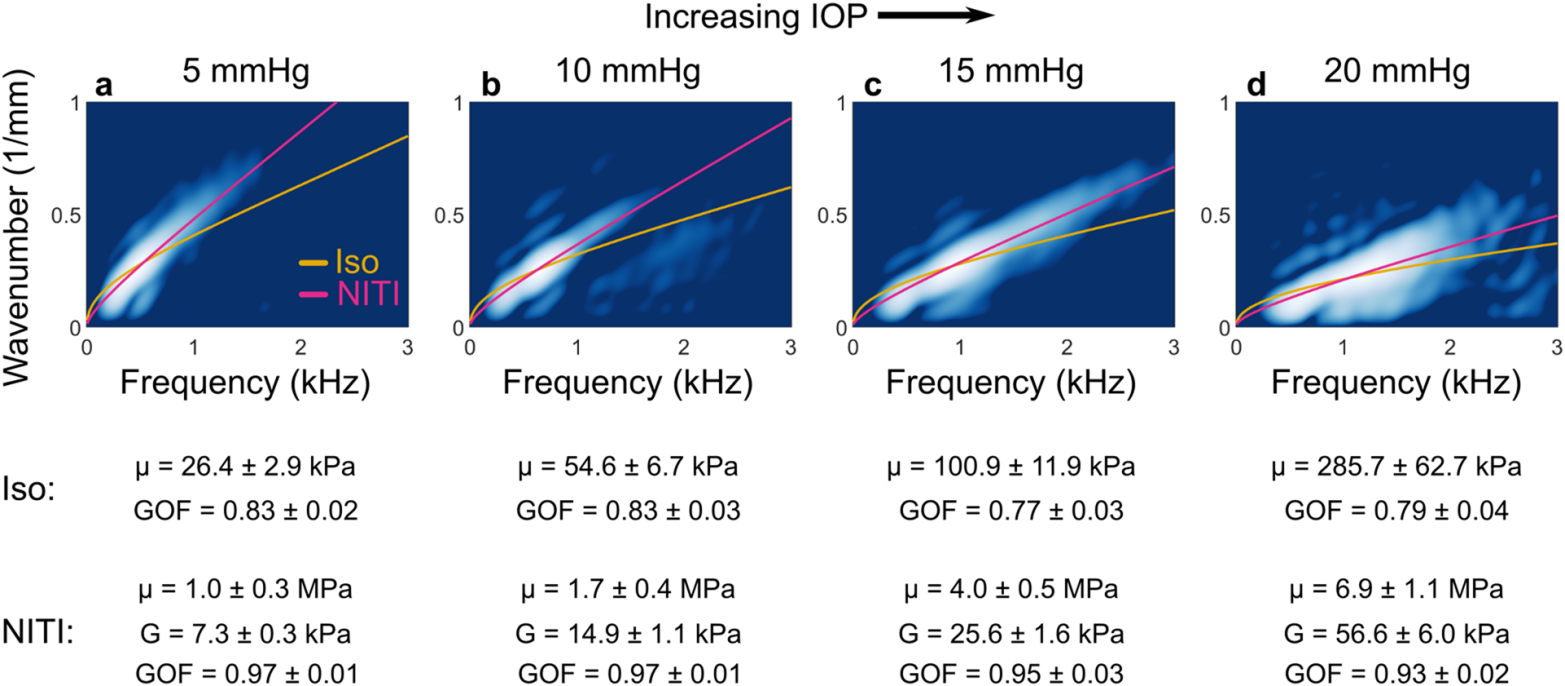
2D Fourier spectra of wave fields generated and tracked with AμT-OCE along the surface of ex vivo porcine cornea at varying intraocular pressure (IOP). At each IOP, the NITI model (pink line) more closely matches the mode behavior compared to the isotropic model (yellow line). Spectra are shown on a log scale over a 20-dB dynamic range at 5 mmHg IOP (**a**), 10 mmHg IOP (**b**), 15 mmHg IOP (**c**), and 20 mmHg IOP (**d**). Parameter estimates and goodness-of-fit (GOF) metrics (mean ± standard deviation, calculated over repeated OCE measurements for a given cornea/IOP) for each model are shown below their corresponding IOP.

In porcine cornea, we fit only the A_0_ mode to both isotropic and NITI dispersion relations. Isotropic fits produced an estimate of the isotropic shear modulus whereas NITI fits produced estimates of both *G* and μ (μ = *E*/3). Using a simplex optimization method (Methods, Supplementary Software), we found dispersion curves that most closely matched the mode structure in 2D spectra. The isotropic model provided a poor fit (Fig. 4f, yellow curve). In contrast, the NITI model closely followed the A_0_ mode (Fig. 4f, pink curve). This trend was consistent for IOP ranging from 5–20 mmHg (Fig. 5).

**Figure 6:**
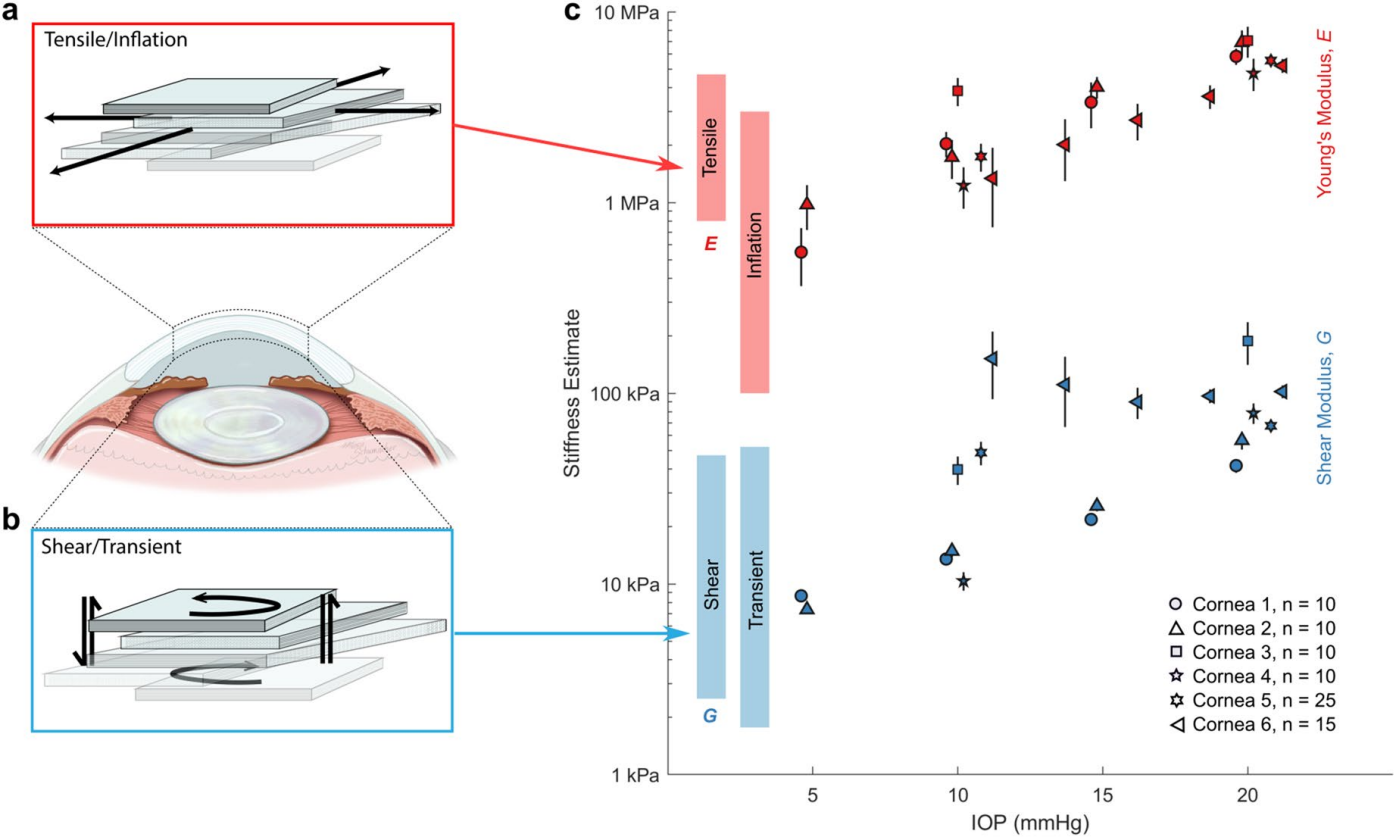
NITI elastic moduli estimates (*G* and μ) obtained from AμT-OCE measurements of porcine cornea. Corneal mechanical response can be divided into (**a**) tensile/inflation and (**b**) shear/transient, which can differ by orders of magnitude. (**c**) Young’s modulus (red markers) and shear modulus (blue markers) estimates were obtained from the NITI model for all porcine corneas over a range of IOP. Marker shape corresponds to six individual porcine corneas. For each cornea sample, 10–25 independent scans were taken at each pressure to minimize system variation. Markers denote the mean modulus estimate from repeated OCE measurements on the same cornea, and error bars denote ± one standard deviation. Modulus estimates correspond closely to the range of values reported in the literature for static inflation/tensile tests (red bars) and shear/transient measurements (blue bars).

Moduli estimates from the NITI model for all corneas and IOPs are summarized in Fig. 6. We observe a multiple order-of-magnitude difference between estimated Young’s modulus (*E*_*TI*_ = 3μ_*TI*_) and the shear modulus *G*_*TI*_, consistent with literature values for static tests and OCE measurements. Both moduli increase with IOP over the observed range; however, the orders-of-magnitude difference in the moduli remains consistent across all IOP.

## Discussion

In dynamic OCE, the cornea is typically considered flat, semi-infinite, and isotropic. These approximations may lead to inaccurate interpretations for real corneal geometry and anatomy. For example, the cornea’s finite thickness and bounded structure were ignored until recently, when comprehensive simulations and measurements showed that guided modes cannot be ignored^52^.

A flat isotropic layer is also approximate because the cornea is curved. To see the influence on guided mode structure, we performed numerical simulations in flat and spherical bounded layers. The numerical model was similar to the flat layer one (“Methods” section), except the domain was a curved isotropic layer of thickness *h* = 0.55 mm with outer radius *R*_*0*_ = 6.5 mm. The axisymmetric solution convolved over a line approximates the AμT source. The wave field and Fourier spectrum along the midline of the propagating wave (measured by AμT-OCE) showed little difference between flat and curved models, suggesting that a flat layer can be a reasonable approximation for cornea. This result is unsurprising as curvature adds propagation speed variations of order *h/R*_*0*_(< 5%)^54,55^. Considering the dramatic effect of corneal anisotropy on measured spectra, curvature can be ignored to first order. Supplementary Note 6 provides a detailed description of this analysis, and the OnScale input file and MATLAB processing functions to reproduce it are available in Supplementary Software.

Corneal microstructure and an array of biomechanical studies strongly suggest that the cornea is transversely isotropic rather than purely isotropic. Recently, Brillouin microscopy was used to probe mechanical anisotropy in the cornea based on angle-dependent measurements of the longitudinal wave speed^44,56^. Using a transversely isotropic model, moduli ${C}_{11}$, ${C}_{13}$, and ${C}_{33}$ were estimated for porcine and human corneas, with values on the order of GPa^44^. This is a promising technology for measuring the longitudinal stiffness coefficients in transversely isotropic tissues, which can provide complementary information to our NITI model and shear-wave dynamic OCE method. Unfortunately, Brillouin microscopy is insensitive to the shear moduli $G={C}_{44}$ and $\mu ={C}_{66}$, which dominate the deformational behavior of NITI tissues. Anisotropy in the longitudinal terms is a second-order effect (Supplementary Note 4), and accurate estimates of $G$ and $\mu$ will likely be essential in driving both clinical measurements of ocular biomechanics and computational models of corneal deformations.

Here we have shown that a NITI model more accurately characterizes elastic waves in dynamic OCE studies of the cornea. In particular, elastic waves measured in cornea and isotropic PVA phantoms produce markedly different wave fields and spectra (Fig. 4), demonstrating that an isotropic model is not appropriate for cornea.

The NITI model is defined by two shear moduli (*G* and μ), decoupling tensile/inflation responses from shear responses commonly monitored in torsional tests and dynamic OCE measurements. Based on existing literature, the Young’s modulus for cornea is expected to be on the order of MPa, while the shear modulus is on the order of kPa. This is not physically possible for isotropic materials.

The Rayleigh wave speed in a NITI material is almost entirely defined by the modulus *G* (see Supplementary Note 3). However, the cornea’s finite thickness produces guided waves depending on both *G* and μ, allowing both parameters to be estimated. Theoretically, the optimal way to determine μ is from the phase velocity spectrum of the S_0_ mode, which is largely defined by μ (Fig. 2c). Unfortunately, numerical simulations and OCE measurements show that excitation at the air/cornea interface transfers little energy to the S_0_ mode, making it nearly impossible to detect. The absence of an S_0_ mode is strong evidence of anisotropy, but also means that the A_0_ mode alone must be used to evaluate both *G* and μ.

Using the proposed NITI model, we estimated decoupled Young’s (*E*_*TI*_ = 3μ_*TI*_) and shear (*G*_*TI*_ ) moduli from measurements of elastic wave propagation in porcine cornea. Results agree well with literature values, with *E*_*TI*_ > 500 kPa and *G*_*TI*_ in the range of 6–200 kPa depending on the IOP (Fig. 6), and generally support the observed orders-of-magnitude difference between the two moduli.

We note that μ shows greater variance relative to *G*. For high anisotropy (μ ≫  *G*), the A_0_ mode is increasingly insensitive to μ. Measurement noise inside the elastic wave bandwidth has a greater effect on μ estimates, and uncertainty increases with increasing μ. Thus, while the multiple order-of-magnitude difference between *G* and μ is accurate, determining the true value of μ requires increasingly higher signal-to-noise ratio as anisotropy increases. This produces large confidence intervals for μ in our measurements. The choice of regularization (used to stabilize the fitting routine, see “Methods” section) can also affect the exact estimate of μ. Consequently, there is far more uncertainty in estimated μ than *G* values, especially for low SNR measurements. Because of this uncertainty and the need to choose a suitable regularization parameter, future work is needed to refine these estimates. Increasing the OCE measurement SNR should improve sensitivity to μ, as described above. Ideally, new methods designed to excite and accurately measure the A_0_ and S_0_ modes together could provide more accurate and sensitive estimates of μ.

**Figure 7:**
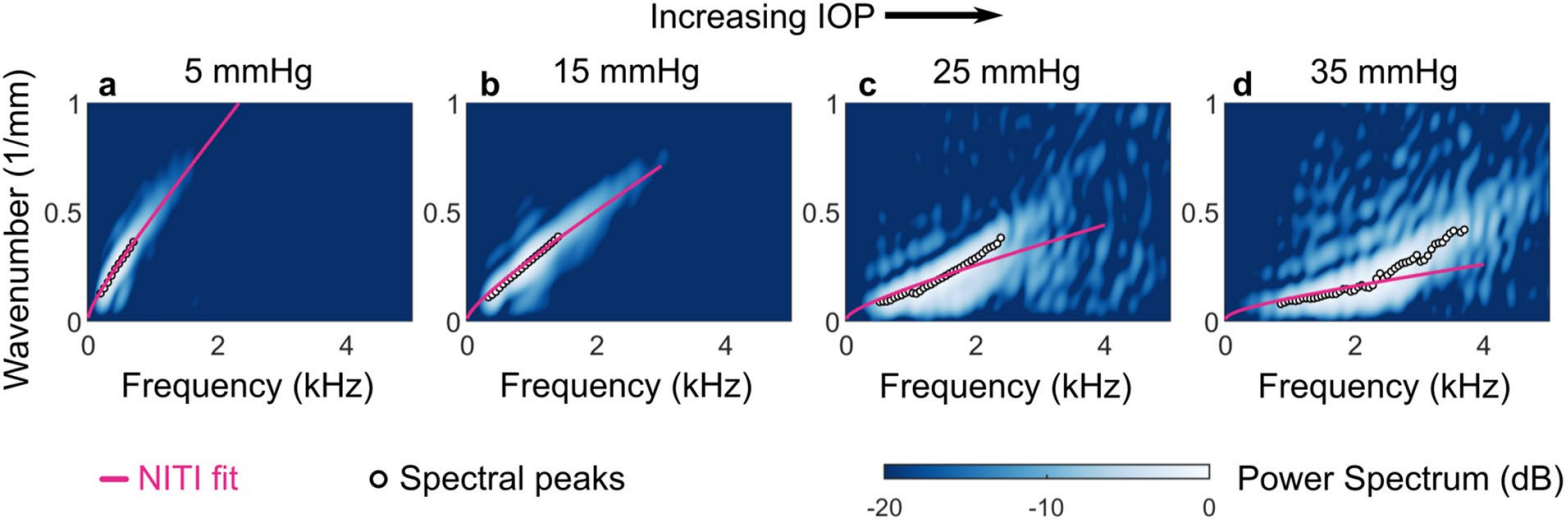
Nonlinear and anisotropic behavior becomes increasingly complex at high intraocular pressure (IOP). 2D Fourier spectra of wave fields generated and tracked with AμT-OCE along the surface of porcine cornea at varying intraocular pressure (IOP) show increasingly complex behavior as IOP increases. Spectra are displayed on a log scale over a 20 dB display dynamic range with spectral peaks (circles) and best-fit dispersion curves for NITI model (pink) at 5 mmHg IOP (**a**), 15 mmHg IOP (**b**), 25 mmHg IOP (**c**), and 35 mmHg IOP (**d**).

It is important to note that the NITI model has limitations. As IOP increases, guided modes change noticeably, particularly at very high IOP. Figure 7 shows two-dimensional spectra and best-fit dispersion curves for a porcine eye measured over a larger IOP range (5, 15, 25, and 35 mmHg). For lower IOP (5 and 15 mmHg), Fourier spectral peaks follow the general A_0_ mode shape, and the NITI model closely fits the data (Fig. 7a,b). However, at higher IOP (25 and 35 mmHg), mode shape changes dramatically, and the NITI fit no longer describes it well (Fig. 7c,d).

Two factors must be considered in this high IOP regime—nonlinearity and complex anisotropy. The cornea exhibits nonlinear elasticity that changes at approximately 30 mmHg due to a two-stage deformation process^57^. Deformation is governed by a net matrix response at low strain and collagen stiffness at higher strain. Changes in load-bearing characteristics at high IOP may dramatically change wave propagation and/or induce more complex anisotropic behavior.

Stress–strain testing and polarization-sensitive imaging of collagen alignment suggest that the cornea exhibits a relatively symmetric tensile response at low strain^38^. As strain increases, fiber orientation changes. Transient elastography studies have also observed in-plane anisotropy in the cornea starting at 15–20 mmHg and increasing with IOP^22,28,35,36,39^. This suggests that the cornea becomes more nonlinear and anisotropic at high IOP, and the NITI model no longer adequately describes it. Complex anisotropy models, such as orthotropic or fibril-based models, may be required at high IOP.

Further studies are needed to evaluate the NITI model’s clinical utility. As human and porcine cornea differ slightly, it must be tested using OCE measurements on humans. More complex models may also be considered to better estimate corneal stiffness at high IOP. At physiologically relevant IOP, however, we expect the NITI model to perform similarly for human and porcine cornea.

The model’s relative simplicity should also facilitate future clinical trials, as it requires a single non-contact measurement, obtained within seconds, to estimate *G* and μ. While other clinical tools, such as the Ocular Response Analyzer (ORA), have been used clinically to infer both corneal stiffness and IOP^58^^,^ sufficient measurement error^59^^,^ in addition to patient discomfort^60^^,^ hinder widespread use. Further, the relationship between in vivo applanation and corneal Young’s Modulus remains unclear^61,62^. The non-contact nature and consistency of AμT-driven OCE strongly support its potential as a practical clinical tool to evaluate corneal elasticity, monitor glaucoma, and study corneal response to ophthalmic interventions.

Non-contact clinical assessment of cornea biomechanics may enable in vivo clinical trials that can provide further insight into the role of biomechanics in cornea function. AμT-OCE can produce accurate maps of mechanical properties, providing reliable, non-contact assessment of corneal biomechanics. Such features make it a potentially valuable tool to evaluate cornea in vivo and to develop future procedures to improve vision by modifying tissue microstructure.

## Methods

### Analytic solution

To derive an analytic solution for guided waves in the cornea, we consider an infinite NITI layer of thickness *h* and density ρ bounded above by air and below by water. The stiffness tensor contains material constants λ, μ, and *G*. We assume a plane strain state, consistent with the pseudo-line source generated in our AμT experiments. Here, we briefly overview the derivation for the guided wave solution. Supplementary Note 4 provides a complete derivation. For a NITI material, the elastic wave equations take the following dimensionless form:

$${u}_{tt}=\left(\frac{\lambda +2\mu }{\mu }\right){u}_{xx}+\left(\frac{G}{\mu }\right){u}_{zz}+\left(\frac{\lambda +G}{\mu }\right){v}_{xz},$$

$${v}_{tt}=\left(\frac{G}{\mu }\right){v}_{xx}+\left(\frac{\lambda +2\mu }{\mu }\right){v}_{zz}+\left(\frac{\lambda +G}{\mu }\right){u}_{xz},$$

where *u* and *v* are the *x*- and *z*-components of the displacement, respectively, and subscripts denote partial differentiation. Assuming harmonic plane wave solutions for displacements in the elastic wave equations for the tissue layer and harmonic acoustic wave solutions for the bounding fluid leads to a secular equation that can be solved for the guided wave frequency-wavenumber dispersion relation (Eq. S4.25^,^Supplementary Note 4).

### Numerical simulation

We developed a finite element numerical model of guided wave propagation in a NITI layer using OnScale (OnScale, Redwood City, CA)^63^. Supplementary Note 5 provides a full description of the model. Briefly, we model the cornea as a thin elastic layer of thickness *h* = 0.55 mm and density ρ = 1,000 kg/m^3^, bounded above by air (free surface condition) and below by a layer of water (modeled as an isotropic solid with density 1,000 kg/m^3^, shear wave speed 0 m/s, and a longitudinal wave speed roughly matched to the solid layer). The outer boundaries of the computational domain were set to absorbing conditions. A pressure load was applied to the air-tissue interface with a Gaussian profile in space and super-Gaussian profile in time. The spatial full-width-at-half-max (FWHM) was 600 μm. It was measured with a needle hydrophone (HNC-1000, Onda, Sunnyvale, CA, USA) in air sampling along a 45° line through the AμT transducer focus. The temporal FWHM was 100 μs, also chosen to match AμT experiments.

The computational domain was discretized using linear finite elements on a regular rectangular grid with at least 40 elements per elastic wavelength. Simulations were solved using explicit time stepping, and the vertical velocity component was extracted for analysis, similar to OCE experiments where only this component is available. Velocity data were directional and band-pass filtered using the same processing as OCE experiments to remove reverberations from faster wave components.

### AμT-OCE to track mechanical waves

To generate elastic waves, we excited samples with acoustic micro-tapping (AμT), a technique using a cylindrically focused, air-coupled ultrasound transducer to induce a localized radiation force at the sample surface^22,64^. The AμT transducer effectively applied a line load to the surface over a wide region relative to the propagation distance of interest, resulting in approximately planar elastic waves (normal to the OCT image plane). The transducer’s full-width-at-half-maximum lateral focus was measured as 420 µm. In the phantom experiment, the spatial push width was approximately 600 µm due to its tilt angle relative to the sample^22^. Due to corneal geometry, the axial focus in the porcine experiment was closer to the theoretically measured axial width of 420 µm^53^.

For AμT (as for laser excitation of ultrasound)^65^^,^ when an elastic wave is excited by an infinitesimally short (in time) push, the spectral characteristics of the wave are defined by the spatial width of the push. In practice, this pressure confinement can be realized when the push duration is shorter than the shear wave propagation time across the excitation zone. Taking this into account, we utilized a 100 µs pulse duration to generate broadband (up to 4 kHz) mechanical waves and induce tissue displacements on the order of hundreds of nanometers. Because the shear wave excitation is reflection-based, most of the acoustic energy (99.9%) is reflected from the sample surface, leading to acoustic exposures well below the limits for ophthalmic applications^22,66^.

The axial particle vibration velocity of propagating mechanical waves was detected using a phase-sensitive frequency-domain OCT (PhS-OCT) system, which has been described in a previous study^53,67–69^. The M-B mode PhS-OCT system includes a broadband super-luminescent diode with a 1,310 nm center wavelength and 86 nm spectral BW (Denselight Ltd., Singapore), a 90/10 beam splitter, a stationary reference arm, a sample arm integrated with a set of galvo-scanners, and a high-speed spectrometer. The incident power on the sample at focus was approximately 8 mW, providing an OCT SNR of 40 dB. This incident power is well within the safety limits for ophthalmic imaging^70^. The sampling rate of the 1,024-pixel line-scan InGaAs array was 46.5 kHz, determining the A-line rate of the system (temporal resolution). The optical resolution was approximately 15 µm axially and 24 µm laterally.

To track mechanical wave propagation on the sample surface, an external TTL trigger synchronized the PhS-OCT system with wave excitation for each M-scan. All data were collected in an M-B format in which 512 A-scans are repeated in the same location (M-scan) at 256 different horizontal locations (B-scan) across the imaging plane (dx = 54.7 µm), forming a complete M-B scan (1,024 depth × 256 lateral locations × 512 temporal frames) with an effective imaging range of 1.5 mm × 10 mm (axial × lateral). One full M-B scan took 3.66 s.

The resulting three-dimensional dataset was then used to reconstruct the propagating wave based on the OCT-measured local particle vibration velocity. The axial vibration velocity at a given location (${v}_{z}\left(x,z,t\right))$ was obtained from the optical phase difference $\Delta {\varphi }_{opt}\left(x,z,t\right)$ between two consecutive A-line scans at each location using the following equation^56^:

$$\begin{array}{c}{v}_{z}\left(x,z,t\right)= \frac{\Delta {\varphi }_{opt}\left(x,z,t\right)\stackrel{-}{\lambda }}{4\pi \stackrel{-}{n}{f}_{s}^{-1}}\end{array}$$

where $\stackrel{-}{\lambda }$ was the center wavelength of the broadband light source, $\stackrel{-}{n}$ was the refractive index of the medium, and ${f}_{s}$ was the sampling frequency. The refractive index in the cornea and phantom were assumed to be 1.38. The system was able to reliably detect displacements greater than ≈ 5 nm.

The surface signal was measured by automatic detection of the sample surface using an edge detection algorithm. Phase data in a 183 μm window below the surface were then extracted and averaged, weighted using one half of a Gaussian window (HWHM = 90 μm, weight decreasing with depth).

### Fitting experimental data with the NITI model

Quantitative moduli estimates in bounded materials require a method to determine the dispersion relation most closely matching observed guided wave modes. Here, we performed this analysis in the frequency-wavenumber domain using a simplex optimization method (*fminsearch*, MATLAB, MathWorks, Natick, MA). A complete description of the fitting procedure, including rationale for fitting in the frequency-wavenumber domain, is included in Supplementary Note 7.

The theoretical solution presented in Supplementary Note 4 acted as the forward model for optimization. A number of physical parameters were considered fixed, including the corneal density (1,000 kg/m^3^), corneal longitudinal wave speed (1,540 m/s), and mean corneal thickness (measured from B-mode OCT images). The cornea was bounded from below by water with a density of 1,000 kg/m^3^ and longitudinal wave speed of 1,480 m/s. Because we did not observe the S_0_ mode in corneal measurements, we extracted only the A_0_ mode from the forward model using a mode-tracing routine (similar to Pavlakovic et al.^71^).

A two-dimensional Fourier transform was applied to OCE-measured surface velocity data to generate a normalized power spectrum. An optimization routine based on the simplex method estimated both shear moduli, *G* and μ, by fitting the experimentally obtained 2D spectra with the analytic solution (Eq. S4.25^,^Supplementary Note 4). For each sample, the thickness (*h*) was measured using automated edge detection algorithms applied to the OCT structural image collected at the start of each MB scan sequence. At each iteration, a dispersion relation for the A_0_ mode was computed for the current iterate (*G*_*i*_, μ_*i*_) based on the forward model. The average power within a small 7-point Gaussian window centered on this dispersion curve was computed, and the algorithm updated iterates of *G* and μ to maximize this quantity. A regularization term is included to ensure that the ratio μ/λ remains small, consistent with the nearly-incompressible assumption. To compare isotropic and NITI model fits, a goodness-of-fit (GOF) metric was defined based on the maximum energy at each given frequency. When this goodness-of-fit metric approaches a value of 1, the model’s dispersion curve accurately captures all of the energy of the A_0_ mode (see Supplementary Note 7 for details).

### Isotropic phantom preparation

A homogenous, isotropic, elastic phantom with controllable mechanical properties was created to experimentally measure wave behavior in a thin plate model. It was fabricated using a similar protocol to that described by Kharine et al.^72^ Briefly, polyvinyl alcohol (PVA) (146–186 kDa, > 99% hydrolyzed, CAS: 9002-89-5, Sigma-Aldrich Corp., St. Louis, MO, USA) was added to a 4:1 mixture of dimethylsulfoxide (DMSO, CAS: 67-68-5, EMD Millipore Corp) and water at a concentration of 4 wt%. To tune the phantom’s optical properties, we added 0.025 wt% titanium dioxide nanoparticles. The solution was covered and stirred at a temperature of 95 °C for approximately 1 h until the PVA was completely dissolved. The solution was degassed in a vacuum chamber to remove any air bubbles before casting in a round mold with a radius of 10 cm. Phantom thickness (0.6 mm, measured by OCT) was controlled by the amount of PVA solution poured into the mold and allowed to settle. The mold was stored at − 20 °C for at least 12 h, or until the phantom was completely frozen. The phantom was then thawed at room temperature, completing one freeze–thaw cycle. After casting, phantoms were removed from their molds and placed in a water bath for at least 48 h to allow the DMSO to diffuse out. Prior to imaging, the PVA phantom was suspended on top of water to force asymmetric boundary conditions similar to those of the cornea^52^.

### Porcine cornea samples

Porcine eyes were enucleated immediately after death and stored in physiological saline until imaging. All OCE measurements were performed within 1 h of euthanization. The whole porcine eyeball was placed into a custom-built holder with a hemispherical cup filled with saline-moisturized cotton to provide an in situ environment. The eye globe was oriented cornea side up with the optic axis vertical and aligned with the OCE scanning beam. A 23-gauge needle connected to an infusion reservoir was inserted through the sclera to control intraocular pressure (IOP). The reservoir height was adjusted to maintain IOP between 5 and 35 mmHg. The mean thickness of the cornea samples was 0.71 ± 0.11 mm.

All studies were carried out in accordance with institutional guidelines and regulations for tissue studies. All experimental protocols followed standard operating procedures established by the University of Washington for the use of animal tissue acquired from an abattoir in research studies.

## Supplementary information

Supplementary Information 1.

Supplementary Information 2.

Supplementary Video 1.

Supplementary Software.

## Data Availability

The authors declare that all data from this study are available within the Article and its Supplementary Information. Raw data for the individual measurements are available on reasonable request. In addition, we have included a Supplementary Software Library containing the MATLAB scripts and functions used in this study, as well as the OnScale finite element input files. A detailed description of the functions and scripts is provided in the Supplementary Software Documentation. We also include three example MATLAB data files: (1) Example OCE data from one porcine cornea measurement, (2) example OnScale results for the NITI guided wave model, and (3) example OnScale results for the spherical layer model.
