# Supplementary material for: Nearly-incompressible transverse isotropy (NITI) of cornea elasticity: model and experiments with acoustic micro-tapping OCE
Source: Sci Rep. 2020 Jul 31;10:12983. doi: 10.1038/s41598-020-69909-9 (PMC7395720; doi:10.1038/s41598-020-69909-9)
Supplement: Supplementary file 1 — Supplementary Information 1. [file 41598_2020_69909_MOESM1_ESM.pdf]

Supplementary Notes

Nearly-incompressible transverse isotropy (NITI) of cornea elasticity: model and experiments with acoustic micro-tapping OCE

John J. Pitre Jr.[1*], Mitchell A. Kirby[1*], David S. Li[1,2], Tueng T. Shen[3], Ruikang K. Wang[1,3], Matthew O'Donnell[1], and Ivan Pelivanov[1]

[1.] University of Washington, Department of Bioengineering, Seattle, Washington, United States
[2.] University of Washington, Department of Chemical Engineering, Seattle, Washington, United States
[3.] University of Washington, Department of Ophthalmology, Seattle, Washington, United States

Supplementary Note 1. Table of literature-reported mechanical testing results for ex vivo cornea

The following table summarizes the large variability in reported values of elastic moduli for healthy ex vivo cornea in a number of mammalian species. We include moduli only for the low-strain region where they generally are near their lowest value. Best effort was taken to report values for fresh tissue samples. The reported moduli vary greatly based on the loading method, reconstruction method, and assumed model.

Tissue Type	Loading Condition	Loading Rate	Reconstruction Method	Assumed Model	Young's Modulus (E)	Shear Modulus (μ)
Porcine	15mmHg - 140mmHg Inflation/Displacement	Dynamic	FEM	Hyperelastic nonlinear Ogden material	300kPa[1]	-
Porcine	15mmHg - 30mmHg Inflation/Air-puff	Dynamic	FEM	Generalized Maxwell viscoelastic	2.6 MPa[2]	-
Porcine	15mmHg Inflation/Air-puff	Dynamic	FEM	Hyperelastic Mooney Rivlin	.99-1.59 MPa[3]	-
Porcine	Tensile	Quasi-static	Stress-strain	-	1.15-1.93 MPa[3]	-
Porcine	0mmHg - 40mmHg Inflation/Air-puff	Dynamic	Pressure-Deformation	Thin shell	.1-.3 MPa[4]	-
Porcine	Tensile	Quasi-static	Stress-strain	-	3.193$\pm$1.589 MPa[4]	-
Porcine	.75mmHg - 170mmHg Inflation	Dynamic	Pressure-Deformation	Thin shell	.15-.3 MPa[5]	-
Porcine	Tensile	Quasi-static	Stress-strain	-	.3-1.1 MPa[6]	-
Porcine	20mmHg Inflation/Air-Puff OCE	Dynamic	Modified Rayleigh-Lamb Equation	Isotropic Homogenous Viscoelastic	60 kPa[7]	-
Porcine	15mmHg - 30mmHg Inflation/Air-Puff OCE	Dynamic	Modified Rayleigh-Lamb Equation	Isotropic Homogenous Viscoelastic	41.8-157 kPa[8]	-
Porcine	Compression	Dynamic	Transient compression stress relaxation	Transverse Isotropic Biphasic	5.61$\pm$2.27 kPa (compression)[9] 1.33$\pm$ .51 MPa (tension)[9]	-
Porcine	Compression	Quasi-static	Compressive strain	Transverse Isotropic Biphasic	.65-6.32 MPa[10]	-

Porcine	Oscillatory shear	Dynamic (.01-2 Hz)	Shear strain	-	-	2.5-9 kPa[11]
Porcine	0mmHg - 30mmHg Inflation	Quasi-static	Pressure-Deformation	Linear elastic	2.29$\pm$1.63MPa[12]	-
Porcine	15mmHg - 35mmHg Inflation/Air-puff	Dynamic	FEM	Linear isotropic viscoelastic	25.5 MPa (anterior) .85 MPa (posterior)[13]	-
Porcine	15mmHg Inflation/Air-Puff OCE	Dynamic	Group Velocity	Homogenous linear isotropic	5.9$\pm$.6 kPa[14]	-
Porcine	Tensile	Quasi-static	Stress-strain	-	.8-2.2 MPa[15]	-
Porcine	Tensile	Quasi-static	Stress-strain	-	3.70$\pm$ .24 MPa[16]	-
Human	Oscillatory shear	Dynamic (.01-2 Hz)	Shear strain	-	-	3-15 kPa[11]
Human	Tensile	Quasi-static	Stress-strain	-	.34-4.1 MPa[17]	-
Human	Acoustic Vibration	Dynamic (50-600Hz)	FEM	Linear isotropic viscoelastic	24.8 kPa (anterior) 19.8 kPa (posterior)[18]	-
Human	15mmHg - 35mmHg Inflation/Air-puff	Dynamic	FEM	Linear isotropic viscoelastic	.71MPa[13]	-
Human	Oscillatory shear	Dynamic (.03 Hz)	Shear strain	-	-	38.7$\pm$8.6 kPa[19]
Human	1D Shear	Quasi-static	Shear strain	-	-	10-90 kPa (Nasal-Temporal) 10-150 kPa (Superior-Inferior)[20]
Human	Tensile	Quasi-static	Stress-strain	-	.8-2.6 MPa[15]	-
Human	Tensile	Quasi-static	Stress-strain	-	3.81$\pm$ .40 MPa[16]	-
Human	Tensile	Quasi-static	Stress-strain	-	19.1$\pm$3.5 MPa[21]	-
Human	.75mmHg- 160mmHg Inflation	Dynamic	Pressure-Deformation	Thin shell	.25-3 MPa[22]	-
Human	.75mmHg- 170mmHg Inflation	Dynamic	Pressure-Deformation	Thin shell	.2-.6 MPa[5]	-
Bovine	1mmHg - 10mmHg Inflation/Piezo-shaker motion MRE	Dynamic (300 Hz)	FEM	Linear isotropic	40-185 kPa[23]	-
New Zealand White Rabbit	15mmHg Inflation/Air-puff	Dynamic	FEM	Nonlinear Hyperelastic Mooney Rivlin plus a Prony-series viscoelastic	5 MPa[24]	-
Rabbit	15mmHg Inflation/Air-Puff OCE	Dynamic	FEM	Homogenous linear isotropic	500-800 kPa[25]	-

Supplementary Note 2. Behavior of NITI materials under common mechanical tests

In this study, we show that a nearly incompressible transversely isotropic (NITI) model may explain many of the discrepancies in reported values of corneal Young's modulus. In particular, there is a multiple order-of-magnitude difference between the values reported by tensile/inflation tests and those reported by shear/transient tests. Tensile tests of corneal strips yield values of 800 kPa – 4.7 MPa,[3,4,6,15–17,21] and inflation tests of corneal trephinates yield values of 100 kPa – 3 MPa.[1–5,12,13,22] In contrast, shear (torsional) tests report shear moduli of 2.5 – 47.3 kPa.[11,20,26] This corresponds to a Young's modulus of 7.5 – 142 kPa if one assumes an

isotropic model. Likewise, transient methods such as optical coherence elastography (OCE) report Young's moduli in the range 5.3 – 157 kPa,[8,27] again relying on an isotropic model to convert shear modulus to Young's modulus.

The NITI model has a key property that can explain both regimes of reported moduli – namely, its shear and tensile behavior are decoupled. Mechanical tests that probe these behaviors independently will report greatly different Young's moduli. In fact, shear tests will report an independent parameter unrelated to Young's modulus. The shear behavior is governed by an independent modulus, which we refer to as G. The tensile behavior is governed by the modulus μ, which can be related to the Young's modulus E_{TI} = 3μ. Here, we demonstrate that the relevant stress-strain relations for each test type will depend primarily on one elastic parameter while remaining agnostic to the other. Furthermore, tensile and inflation measurements should yield similar values, as should shear and transient ones.

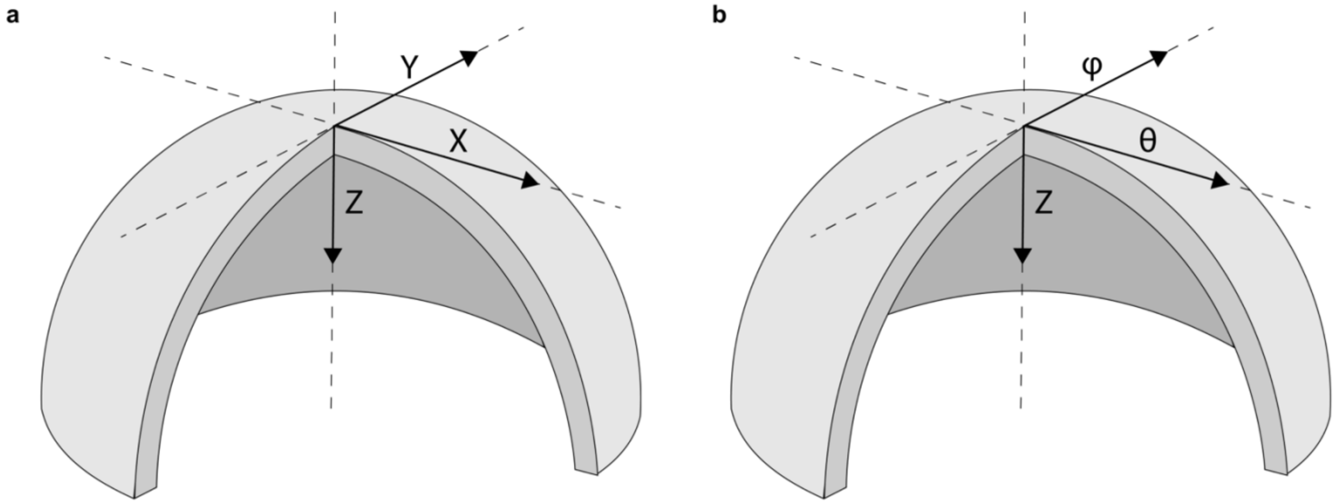

Supplementary Fig. S2. Corneal geometry and local coordinate axes used in analyzing deformation for (a) tensile, torsional, and wave propagation tests and (b) inflation tests.

Hooke's law for a NITI material. Hooke's law relates stress σ and strain ε in a linearly elastic solid. In its most general form, it may be written

$$\sigma_{ij} = c_{ijkl}\varepsilon_{kl}, \quad (S2.1)$$

where summation is implied over repeated indices. In a general anisotropic solid, the fourth-rank stiffness tensor c_{ijkl} contains 21 independent elastic constants. This reduces to 5 constants in a transversely isotropic solid, 3 constants in a NITI solid, and 2 constants in an isotropic solid. For convenience, Equation S2.1 is often written in Voigt notation

$$\begin{bmatrix} \sigma_{xx} \\ \sigma_{yy} \\ \sigma_{zz} \\ \tau_{yz} \\ \tau_{xz} \\ \tau_{xy} \end{bmatrix} = \begin{bmatrix} C_{11} & C_{12} & C_{13} & C_{14} & C_{15} & C_{16} \\ * & C_{22} & C_{23} & C_{24} & C_{25} & C_{26} \\ * & * & C_{33} & C_{34} & C_{35} & C_{36} \\ * & * & * & C_{44} & C_{45} & C_{46} \\ * & * & * & * & C_{55} & C_{56} \\ * & * & * & * & * & C_{66} \end{bmatrix} \begin{bmatrix} \varepsilon_{xx} \\ \varepsilon_{yy} \\ \varepsilon_{zz} \\ \gamma_{yz} \\ \gamma_{xz} \\ \gamma_{xy} \end{bmatrix}, \quad (S2.2)$$

where τ_{ij} denotes shear stresses, $\gamma_{ij} = 2\varepsilon_{ij}$ denotes shear strains, and stars denote symmetric entries (i.e. $C_{21} = C_{12}$). The subscripts x, y, and z refer to standard Cartesian axes, shown for the cornea in Suppl. Fig. S2a.

To define a material as nearly incompressible, we first consider the case of an isotropic medium. The stiffness matrix in this case depends on two constants, the Lamé parameters λ and μ, and the stress-strain relation is

$$\begin{bmatrix} \sigma_{xx} \\ \sigma_{yy} \\ \sigma_{zz} \\ \tau_{yz} \\ \tau_{xz} \\ \tau_{xy} \end{bmatrix} = \begin{bmatrix} \lambda + 2\mu & \lambda & \lambda & & & \\ \lambda & \lambda + 2\mu & \lambda & & & \\ \lambda & \lambda & \lambda + 2\mu & & & \\ & & & \mu & & \\ & & & & \mu & \\ & & & & & \mu \end{bmatrix} \begin{bmatrix} \varepsilon_{xx} \\ \varepsilon_{yy} \\ \varepsilon_{zz} \\ \gamma_{yz} \\ \gamma_{xz} \\ \gamma_{xy} \end{bmatrix}. \tag{S2.3}$$

We then define the mean internal pressure P and the dilatation θ in the medium as

$$P = \frac{1}{3}\left(\sigma_{xx} + \sigma_{yy} + \sigma_{zz}\right), \tag{S2.4}$$

$$\theta = \varepsilon_{xx} + \varepsilon_{yy} + \varepsilon_{zz}. \tag{S2.5}$$

For a nearly-incompressible material, the dilatation must equal zero, but the internal pressure must remain finite. Rewriting the stress tensor in terms of the dilatation, we see that

$$\sigma_{ij} = (\lambda\theta)\delta_{ij} + 2\mu\varepsilon_{ij}, \tag{S2.6}$$

and the internal pressure becomes

$$P = \lambda\theta + \frac{2}{3}\mu\theta. \tag{S2.7}$$

As $\theta \to 0$, the pressure remains finite only if $\lambda \to \infty$. Therefore, the internal pressure for a nearly-incompressible material may be defined

$$P = \lim_{\substack{\lambda\to\infty \\ \theta\to 0}} \lambda\theta\,. \tag{S2.8}$$

A similar analysis helps define conditions for a nearly-incompressible transversely isotropic (NITI) solid.[28] Unlike the isotropic case, a NITI solid cannot be defined precisely by a single condition. Instead, there are a family of materials which satisfy the incompressibility condition, with constraints on valid stiffness values. For a general TI solid, the stress-strain relation is defined by five independent constants (with $C_{12} = C_{11} - 2C_{66}$):

$$\begin{bmatrix} \sigma_{xx} \\ \sigma_{yy} \\ \sigma_{zz} \\ \tau_{yz} \\ \tau_{xz} \\ \tau_{xy} \end{bmatrix} = \begin{bmatrix} C_{11} & C_{12} & C_{13} & & & \\ C_{12} & C_{11} & C_{13} & & & \\ C_{13} & C_{13} & C_{33} & & & \\ & & & C_{44} & & \\ & & & & C_{44} & \\ & & & & & C_{66} \end{bmatrix} \begin{bmatrix} \varepsilon_{xx} \\ \varepsilon_{yy} \\ \varepsilon_{zz} \\ \gamma_{yz} \\ \gamma_{xz} \\ \gamma_{xy} \end{bmatrix}. \tag{S2.9}$$

For notational convenience, we may rename some of the constants as follows

$$\begin{bmatrix}\sigma_{xx}\\ \sigma_{yy}\\ \sigma_{zz}\\ \tau_{yz}\\ \tau_{xz}\\ \tau_{xy}\end{bmatrix}=\begin{bmatrix}\lambda+2\mu & \lambda & C_{13} & & & \\ \lambda & \lambda+2\mu & C_{13} & & & \\ C_{13} & C_{13} & C_{33} & & & \\ & & & G & & \\ & & & & G & \\ & & & & & \mu\end{bmatrix}\begin{bmatrix}\varepsilon_{xx}\\ \varepsilon_{yy}\\ \varepsilon_{zz}\\ \gamma_{yz}\\ \gamma_{xz}\\ \gamma_{xy}\end{bmatrix}. \qquad (S2.10)$$

As before, the dilatation of a material is defined in terms of the normal strain components:

$$\theta=\varepsilon_{xx}+\varepsilon_{yy}+\varepsilon_{zz} \qquad (S2.11)$$

where the strains are related to the normal stresses σ_{xx}, σ_{yy}, and σ_{zz} and the longitudinal part of the compliance tensor C_ℓ^{-1}:

$$\varepsilon_\ell=C_\ell^{-1}\sigma_\ell, \qquad (S2.12)$$

where

$$C_\ell^{-1}=\frac{1}{\Delta}\begin{bmatrix}C_{11}C_{33}-C_{13}^2 & C_{13}^2-C_{12}C_{33} & C_{13}(C_{12}-C_{11})\\ C_{13}^2-C_{12}C_{33} & C_{11}C_{33}-C_{13}^2 & C_{13}(C_{12}-C_{11})\\ C_{13}(C_{12}-C_{11}) & C_{13}(C_{12}-C_{11}) & C_{11}^2-C_{12}^2\end{bmatrix} \qquad (S2.13)$$

$$\Delta=\det C_\ell=(C_{11}-C_{12})[(C_{11}+C_{12})C_{33}-2C_{13}^2] \qquad (S2.14)$$

As before, we require the normal stresses and the mean internal pressure of the material to describe deformations of this system. We define new variables

$$Q_1=C_{13}-\lambda \qquad (S2.15)$$

$$Q_2=C_{33}-(\lambda+2\mu) \qquad (S2.16)$$

and write the stresses, internal pressure P, and dilatation θ as:

$$\sigma_{xx}=\lambda\theta+2\mu\varepsilon_{xx}+Q_1\varepsilon_{zz} \qquad (S2.17)$$

$$\sigma_{yy}=\lambda\theta+2\mu\varepsilon_{yy}+Q_1\varepsilon_{zz} \qquad (S2.18)$$

$$\sigma_{zz}=\lambda\theta+2\mu\varepsilon_{xx}+Q_1(\varepsilon_{xx}+\varepsilon_{yy})+Q_2\varepsilon_{zz} \qquad (S2.19)$$

$$P=\lambda\theta+\frac{2}{3}\mu\theta+\frac{1}{3}Q_1\theta+\frac{1}{3}(Q_1+Q_2)\varepsilon_{zz} \qquad (S2.20)$$

$$\theta=\frac{2\mu}{\Delta}\{(Q_2-Q_1+2\mu)(\sigma_{xx}+\sigma_{yy})-2(Q_1-\mu)\sigma_{zz}\} \qquad (S2.21)$$

$$\Delta=4\mu\left\{(\lambda+\mu)Q_2-Q_1(Q_1+2\lambda)+3\mu\left(\lambda+\frac{2}{3}\mu\right)\right\} \qquad (S2.22)$$

We define a NITI material as one for which the internal pressure remains finite as $\lambda \to \infty$ and $\theta \to 0$, while the parameters μ, Q_1, and Q_2 remain finite. Note that we can require C_{13} and C_{33} to be asymptotically equal to λ so that Q_1 and Q_2 remain finite.

To satisfy incompressibility exactly for any general stress field requires that the coefficients of the stresses equal zero exactly:

$$Q_2 - Q_1 + 2\mu = 0 \tag{S2.23}$$

$$Q_1 - \mu = 0 \tag{S2.24}$$

which yields $Q_1 = \mu$, $Q_2 = -\mu$. These values correspond to the case $\Delta = 0$, for which the stiffness matrix is singular. This means that there is no exact general definition of a NITI material. However, from the definition of θ, we note that $\theta \to 0$ if $\Delta \to \infty$, provided that $2\mu\{(Q_2 - Q_1 + 2\mu)(\sigma_{xx} + \sigma_{yy}) - 2(Q_1 - \mu)\sigma_{zz}\}$ remains finite (it does by definition). We note that in the limit $\lambda \to \infty$, this condition is indeed satisfied.

As in the isotropic case, the definition of the internal pressure for a NITI material requires that $\lim_{\lambda\to\infty} \lambda\theta$ must remain finite:

$$\lim_{\lambda\to\infty} \lambda\theta \; = \lim_{\lambda\to\infty} \frac{2\mu\lambda}{\Delta}\{(Q_2 - Q_1 + 2\mu)(\sigma_{xx} + \sigma_{yy}) - 2(Q_1 - \mu)\sigma_{zz}\}. \tag{S2.25}$$

Note that the term in braces is finite, and we may evaluate the limit as follows:

$$\lim_{\lambda\to\infty} \lambda\theta = 2\mu\{(Q_2 - Q_1 + 2\mu)(\sigma_{11} + \sigma_{22}) - 2(Q_1 - \mu)\sigma_{33}\} \cdot \lim_{\lambda\to\infty} \frac{\lambda}{\Delta}$$

$$\lim_{\lambda\to\infty} \frac{\lambda}{\Delta} = \frac{\lambda}{4\mu\{\lambda(Q_2 - 2Q_1 + 3\mu) + \mu Q_2 - Q_1^2 + 2\mu^2\}}$$

$$\lim_{\lambda\to\infty} \frac{\lambda}{\Delta} = \frac{1}{4\mu(Q_2 - 2Q_1 + 3\mu)} \tag{S2.26}$$

which is finite provided $Q_2 - 2Q_1 + 3\mu \neq 0$.

The conditions for a NITI material then may be summarized as:

1. C_{13} and C_{33} are asymptotically equal to λ $\quad (S2.27a)$
2. $\Delta \neq 0$ $(C_{13} \neq C_{33})$ $\quad (S2.27b)$
3. $Q_2 - 2Q_1 + 3\mu \neq 0$ $\quad (S2.27c)$

Note that these are very loose conditions. For instance, given two constants A and B that are small relative to λ, we then note that

$$C_{13} = \lambda + A \tag{S2.28}$$

$$C_{33} = \; \lambda + B \tag{S2.29}$$

satisfy condition $(S2.27a)$.

These give $Q_1 = A$ and $Q_2 = B + 2\mu$. Of course, there are still values of A and B that violate the constraints, but there are at most three such real values of A for any given B:

$$\Delta = 4\mu\left\{(\lambda+\mu)(B+2\mu) - A(A+2\lambda) + 3\mu\left(\lambda + \frac{2}{3}\mu\right)\right\}, \tag{S2.30}$$

$$B - 2A + 5\mu \neq 0 \tag{S2.31}$$

So there is an extensive family of NITI materials, but we must be careful of the relative values of the stiffness entries. This is especially problematic for an automated optimization routine that must avoid these singular points when inverting the stiffness values from observed mechanical behavior.

The simplest solution is to define a NITI material by taking the longitudinal portion of the stiffness matrix to be isotropic. In this case, $C_{13} = \lambda$ and $C_{33} = \lambda + 2\mu$. This is equivalent to $Q_1 = Q_2 = 0$. In this case, we have

$$\Delta = 12\mu^2\left(\lambda + \frac{2}{3}\mu\right) \tag{S2.32}$$

which is clearly nonzero. Additionally, $Q_2 - 2Q_1 + 3\mu \neq 0$ is obviously satisfied. For a NITI material, Equation S2.2 therefore reduces to

$$\begin{bmatrix}\sigma_{xx}\\ \sigma_{yy}\\ \sigma_{zz}\\ \tau_{yz}\\ \tau_{xz}\\ \tau_{xy}\end{bmatrix} = \begin{bmatrix}\lambda+2\mu & \lambda & \lambda & & & \\ \lambda & \lambda+2\mu & \lambda & & & \\ \lambda & \lambda & \lambda+2\mu & & & \\ & & & G & & \\ & & & & G & \\ & & & & & \mu\end{bmatrix}\begin{bmatrix}\varepsilon_{xx}\\ \varepsilon_{yy}\\ \varepsilon_{zz}\\ \gamma_{yz}\\ \gamma_{xz}\\ \gamma_{xy}\end{bmatrix}. \tag{S2.33}$$

This approximation corresponds to the case of a weakly anisotropic material, since the only anisotropy is due to the shear terms $C_{44} = G$. It was recently demonstrated using Brillouin microscopy that the cornea is only weakly anisotropic in its longitudinal terms, with stiffness values reported as C_{11} = 2.971 GPa, C_{33} = 2.662 GPa, and C_{13} ≈ 2.680 GPa.[29] These are clearly not as restrictive as our assumption, but in practice, these terms contribute little to the behavior we probe (guided waves) since it is dominated by the shear terms. We demonstrate this in Supplementary Note 4.

In the following sections, we consider the stress-strain behavior of the NITI model (Equation S2.33) under various corneal mechanical tests.

Tensile testing of corneal strips. In tensile tests, rectangular strips of ex vivo cornea are subjected to uniaxial tension. The corresponding strain is measured, and the Young's modulus is quantified as $E = \sigma/\varepsilon$. In general, the orientation of the cornea strip lies in the *xy*-plane, and the direction of the applied stress aligns with the long axis of the strip. While anisotropy within the *xy*-plane has been reported by some studies, the degree of anisotropy at low IOP (low pre-stress)[6,30–33] suggests that the cornea microstructure can be approximated with the NITI model as symmetric for any direction in the *xy*-plane. Thus, it is sufficient to consider only one orientation of the strip. Consider uniaxial loading in the x direction. The stress-strain relation becomes

$$\begin{bmatrix}\sigma_{xx}\\0\\0\\0\\0\\0\end{bmatrix}=\begin{bmatrix}\lambda+2\mu & \lambda & \lambda & & & \\ \lambda & \lambda+2\mu & \lambda & & & \\ \lambda & \lambda & \lambda+2\mu & & & \\ & & & G & & \\ & & & & G & \\ & & & & & \mu\end{bmatrix}\begin{bmatrix}\varepsilon_{xx}\\\varepsilon_{yy}\\\varepsilon_{zz}\\\gamma_{yz}\\\gamma_{xz}\\\gamma_{xy}\end{bmatrix}. \quad (S2.34)$$

It is clear from this linear system that the shear strains are zero and consequentially any equations with G vanish. We are left with

$$\begin{bmatrix}\sigma_{xx}\\0\\0\end{bmatrix}=\begin{bmatrix}\lambda+2\mu & \lambda & \lambda\\ \lambda & \lambda+2\mu & \lambda\\ \lambda & \lambda & \lambda+2\mu\end{bmatrix}\begin{bmatrix}\varepsilon_{xx}\\\varepsilon_{yy}\\\varepsilon_{zz}\end{bmatrix}. \quad (S2.35)$$

Solving the third row for ε_{zz} gives

$$\varepsilon_{zz}=-\frac{\lambda}{\lambda+2\mu}\left(\varepsilon_{xx}+\varepsilon_{yy}\right). \quad (S2.36)$$

Substituting this into the second row and solving for ε_{yy} gives

$$\varepsilon_{yy}=-\frac{\lambda}{2(\lambda+\mu)}\varepsilon_{xx}. \quad (S2.37)$$

Finally, substituting into the first row and defining the Young's modulus as $E=\sigma_{xx}/\varepsilon_{xx}$, we find

$$E=\frac{\mu(3\lambda+2\mu)}{\lambda+\mu}, \quad (S2.38)$$

which in the incompressible limit $\lambda\to\infty$ gives $E = 3\mu$. From this, we see that a NITI material under tensile test will have an apparent Young's modulus that depends only on μ and not on G.

Inflation tests of corneal trephinates. In corneal inflation tests, a circular region of the cornea and sclera (called a trephinate) is dissected from an ex vivo eye (see for example, Anderson et. al.[1]). It is clamped above a fluid-filled chamber and sealed around the scleral rim. The chamber is connected to a water column whose height is varied to simulate intraocular pressure (IOP). For a given pressure p, the test measures the rise of the corneal apex r. The corneal thickness h, radius of curvature R, and the contact angle at the clamped edge γ are also estimated. Deformation of the cornea is then modeled using the theory of spherical shells.[34]

The corneal inflation test can be described by the coordinate system shown in Suppl. Fig. S2b. The deformation of an infinitesimal shell element under pressure is governed by the normal stress resultants N_φ and N_θ, bending moments M_φ and M_θ, and shear stress resultant Q_φ. Here, φ represents the meridional coordinate (sometimes denoted by y in local element coordinates) and θ denotes the azimuthal coordinate (sometimes denoted as x in local element coordinates). The resultants and moments are defined:

$$N_\theta=\int_{-\frac{h}{2}}^{\frac{h}{2}}\sigma_\theta\left(1-\frac{z}{R}\right)\mathrm{d}z\,, \quad (2.39)$$

$$N_\varphi=\int_{-h/2}^{h/2}\sigma_\varphi\left(1-\frac{z}{R}\right)\mathrm{d}z,$$

$$M_\theta = \int_{-\frac{h}{2}}^{\frac{h}{2}} \sigma_\theta z \left(1 - \frac{z}{R}\right) \mathrm{dz}\,,$$

$$M_\varphi = \int_{-h/2}^{h/2} \sigma_\varphi z \left(1 - \frac{z}{R}\right) \mathrm{dz},$$

$$Q_\varphi = \int_{-h/2}^{h/2} \tau_{\varphi z} \left(1 - \frac{z}{R}\right) \mathrm{dz}.$$

Balancing the forces and moments on the shell element and considering the relationship between strain and the deformed shape leads to a system of five equations describing the rise of the corneal apex following inflation.[1]

In assigning a constitutive model to the shell, we apply Hooke's law in the local coordinate system of the shell element. As the shell is assumed to be thin relative to its radius of curvature ($h \ll R$), a plane stress approximation is also applied. This yields the following stress-strain relation for the shell element:

$$\begin{bmatrix} \sigma_\theta \\ \sigma_\varphi \\ 0 \\ 0 \\ 0 \\ \tau_{\theta\varphi} \end{bmatrix} = \begin{bmatrix} \lambda + 2\mu & \lambda & \lambda & & & \\ \lambda & \lambda + 2\mu & \lambda & & & \\ \lambda & \lambda & \lambda + 2\mu & & & \\ & & & G & & \\ & & & & G & \\ & & & & & \mu \end{bmatrix} \begin{bmatrix} \varepsilon_\theta \\ \varepsilon_\varphi \\ \varepsilon_z \\ \gamma_{\varphi z} \\ \gamma_{\theta z} \\ \gamma_{\theta\varphi} \end{bmatrix}. \tag{S2.40}$$

We immediately see, as with the tensile test, that the shear strains vanish and the solution will not depend on G. Proceeding as in the tensile test, we solve the third row for ε_z,

$$\varepsilon_z = -\frac{\lambda}{\lambda + 2\mu} \left(\varepsilon_\theta + \varepsilon_\varphi\right) \tag{S2.41}$$

and substitute into rows 1 and 2 to obtain a simplified system

$$\sigma_\theta = \frac{4\mu(\lambda + \mu)}{\lambda + 2\mu} \varepsilon_\theta + \frac{2\lambda\mu}{\lambda + 2\mu} \varepsilon_\varphi, \tag{S2.42}$$

$$\sigma_\varphi = \frac{2\lambda\mu}{\lambda + 2\mu} \varepsilon_\theta + \frac{4\mu(\lambda + \mu)}{\lambda + 2\mu} \varepsilon_\varphi. \tag{S2.43}$$

For convenience, we convert Equations S2.42 and S2.43 from λ-μ (Lamé parameter) form to the Young's modulus and Poisson's ratio form using the following relationships:

$$\lambda = \frac{E\nu}{(1 + \nu)(1 - 2\nu)}, \tag{S2.44}$$

$$\mu = \frac{E}{2(1 + \nu)}. \tag{S2.45}$$

to obtain

$$\sigma_\theta = \frac{E}{1 - \nu^2} \left(\varepsilon_\theta + \nu\varepsilon_\varphi\right), \tag{S2.46}$$

$$\sigma_\varphi = \frac{E}{1 - \nu^2} \left(\varepsilon_\varphi + \nu\varepsilon_\theta\right). \tag{S2.47}$$

For an incompressible material, ν = 0.5, and the stresses depend only on the Young's modulus E = 3μ. As these stresses are the only terms that appear in the stress resultants and bending moments, it follows that those quantities depend only on μ. The apical rise measured in an inflation test depends on these resultants and moments, and so also depends only on μ. Thus, like the tensile test, an inflation test of a NITI material will measure μ and not G.

Shear torsional test of corneal trephinates. In torsional tests of corneal trephinates, a cylindrical section of ex vivo cornea (with thickness h and radius R) is placed between parallel platens and a rotational deformation Θ is applied to the sample. The torque T at the top platen is measured, and Hooke's law for shear is used to estimate the shear modulus of the sample. The torque is given by the integral

$$T = \int_0^R \int_0^{2\pi} \tau_{\theta z} r \, \mathrm{d}\theta \mathrm{d}r \, . \qquad (S2.48)$$

The transformation from Cartesian coordinates to cylindrical coordinates gives the shear stress and strain

$$\tau_{\theta z} = -\sin\theta \, \tau_{xz} + \cos\theta \, \tau_{yz}, \qquad (S2.49)$$

$$\gamma_{\theta z} = -\sin\theta \, \gamma_{xz} + \cos\theta \, \gamma_{yz}. \qquad (S2.50)$$

For the NITI material, we have

$$\tau_{xz} = G\gamma_{xz}, \qquad (S2.51)$$

$$\tau_{yz} = G\gamma_{yz}. \qquad (S2.52)$$

Substituting equations S2.51 and S2.52. into S2.49, we obtain

$$\tau_{\theta z} = G\gamma_{\theta z}. \qquad (S2.53)$$

The shear strain and stress can be approximated as

$$\gamma_{\theta z} = \frac{r\Theta}{h} \qquad (S2.54)$$

$$\tau_{\theta z} = \frac{Gr\Theta}{h} \qquad (S2.55)$$

Evaluating the torque integral and solving for G, we obtain

$$G = T\frac{2h}{\pi\Theta R^4}. \qquad (S2.56)$$

Clearly, the shear torsional test provides an estimate of G only and is not influenced by μ. Thus, modulus estimates from shear torsional tests may vary greatly from tensile and inflation measurements.

Dynamic measurements of corneal elasticity. Dynamic or transient elasticity measurements use the propagation of elastic waves to estimate elastic moduli. They can provide non-destructive in vivo measurements of corneal elasticity and have the potential to be useful in clinical measurements. Because soft biological tissues are nearly incompressible, the longitudinal wave speed is large (≈1540 m/s) compared to the shear wave speed (1-10 m/s), and the shear wave speed provides a full description of tissue elasticity.

Two models of elastic wave propagation have been used to estimate corneal Young's modulus. The simplest of these treats the cornea as a semi-infinite isotropic solid.[27] This model suffers from serious inaccuracies in

converting the measured group velocity to bulk shear wave speed.[35] However, we analyze the assumption here for completeness. In this case, a Rayleigh wave propagates along the air-cornea interface at constant speed proportional to the bulk shear wave speed. By measuring the group velocity of the Rayleigh wave c_R, one can estimate the Young's modulus as

$$E = 3\rho\left(\frac{c_R}{0.9553}\right)^2. \qquad (S2.57)$$

In a transversely isotropic material, the Rayleigh wave speed can be obtained numerically by employing the Stroh formalism[36–40] or evaluating the Green's function.[41] For materials with $G < \mu$, such as we expect for the cornea, the Rayleigh wave speed is primarily governed by G and only slightly influenced by μ (Supplementary Note 3 provides detailed analysis of the Rayleigh wave speed in a NITI medium).

The cornea should be more accurately modeled as a bounded material. The bounded geometry gives rise to dispersive guided waves, whose frequency-wavenumber behavior must be analyzed to quantify elasticity. Partial wave analysis of the cornea as a flat isotropic plate bounded above by air and below by water leads to a secular equation that describes guided wave modes.[8,42] We detail the mode behavior (Results, Guided wave behavior in a bounded NITI layer) and the partial wave solution for a NITI material (Supplementary Note 4) elsewhere in this work.

In both our study and previous OCE studies of porcine cornea, only the A_0 mode is analyzed to estimate elasticity. For a bounded NITI material, the A_0 mode is governed primarily by G and slightly influenced by μ. Therefore, in both shear torsional tests and dynamic OCE tests, the observed mechanical behavior is governed primarily by G rather than μ (as in tensile/inflation tests). This is consistent with shear modulus values reported by dynamic OCE studies where guided wave behavior has been taken into account through dispersion analysis of the A_0 mode.[8,30]

Supplementary Note 3. Wave behavior in a bulk NITI medium

Transversely isotropic materials support three bulk waves (quasi-longitudinal, quasi-shear, and shear) whose wave speeds depend on the propagation angle. The mechanical behavior is symmetric with respect to rotations about the *z*-axis, but varies with respect to the angle θ formed by the propagation direction and the *z*-axis (Suppl. Fig. S3.1a).

When the propagation direction lies in the *xy*-plane (θ = 90°), three pure wave modes exist (one longitudinal and two orthogonal shear waves). Their wave speeds are

$$c_L = \sqrt{\frac{C_{11}}{\rho}} \qquad (S3.1)$$

$$c_{S1} = \sqrt{C_{44}/\rho}, \text{ polarized in } z,$$

$$c_{S2} = \sqrt{C_{66}/\rho}, \text{ polarized in the } xy\text{-plane},$$

where ρ is the material density.

For a general propagation angle, the polarization of propagating waves is not fully parallel or orthogonal to the propagation direction and, therefore, one quasi-longitudinal, one quasi-shear, and one shear wave should be considered. The following equations can be used to calculate their wave speeds [43]:

$$c_{qL} = \sqrt{\frac{C_{11}\sin^2\theta + C_{33}\cos^2\theta + C_{44} + \sqrt{M(\theta)}}{2\rho}}, \tag{S3.2}$$

$$c_{qS} = \sqrt{\frac{C_{11}\sin^2\theta + C_{33}\cos^2\theta + C_{44} - \sqrt{M(\theta)}}{2\rho}}, \tag{S3.3}$$

$$c_{S} = \sqrt{\frac{C_{66}\sin^2\theta + C_{44}\cos^2\theta}{2\rho}}, \tag{S3.4}$$

$$M(\theta) = [(C_{11} - C_{44})\sin^2\theta + (C_{44} - C_{33})\cos^2\theta]^2 + (C_{13} + C_{44})^2\sin^2 2\theta\,. \tag{S3.5}$$

For the NITI model, these equations simplify to

$$c_{qL} = \sqrt{\frac{\lambda + 2\mu + G + \sqrt{M(\theta)}}{2\rho}}, \tag{S3.6}$$

$$c_{qS} = \sqrt{\frac{\lambda + 2\mu + G - \sqrt{M(\theta)}}{2\rho}}, \tag{S3.7}$$

$$c_{S} = \sqrt{\frac{\mu\sin^2\theta + G\cos^2\theta}{2\rho}}, \tag{S3.8}$$

$$M(\theta) = (\lambda + 2\mu - G)^2\cos^2 2\theta + (\lambda + G)^2\sin^2 2\theta\,. \tag{S3.9}$$

By evaluating these equations for a range of angles using approximate mechanical properties for cornea (ρ = 1000 kg/m^3, μ = 1 MPa, G = 20 kPa, and setting λ so that the average longitudinal wave speed is approximately 1540 m/s), we can gain some intuition of the bulk wave behavior in a NITI material. The quasi-longitudinal wave speed is nearly constant over all angles, with variations of ± 0.01%. The quasi-shear waves' speeds show a large range of variability over angle (Suppl. Fig. S3.1b).

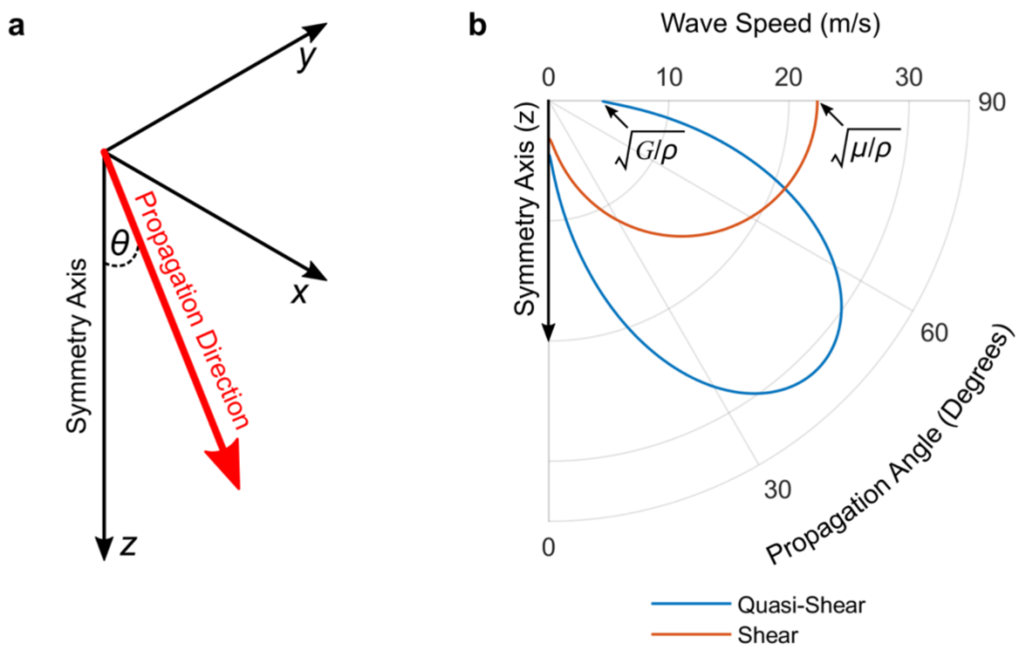

Supplementary Fig. S3.1. (a) Coordinate system, symmetry axis, and propagation angle for a TI material. (b) Quasi-shear and shear wave speed as a function of propagation angle for a NITI material with properties representative of cornea (ρ = 1000 kg/m^3, μ = 1 MPa, G = 20 kPa, λ = 2.37 GPa).

When the xy-plane corresponds to a free surface, a Rayleigh wave propagates along the surface with speed c_R. The value of c_R can be found using the Stroh formalism.[36–40] Here, we consider the solution of a Rayleigh wave propagating in the $\mathbf{m}$ = [1, 0, 0] direction along the free surface defined by normal vector $\mathbf{n}$ = [0, 0, 1] (because of symmetry in the xy-plane, the propagation direction is arbitrary). The Stroh formalism defines the 3×3 matrices

$$Q_{ik} = c_{ijkl}m_j m_l - \rho v^2 \delta_{ik} \quad (S3.10)$$

$$R_{ik} = c_{ijkl}m_j n_l \quad (S3.11)$$

$$T_{ik} = c_{ijkl}n_j n_l \quad (S3.12)$$

where c_{ijkl} is the fourth-order stiffness tensor, ρ is the density, and v is the wave speed. These matrices are combined to form the 6×6 Stroh eigenvalue problem,

$$\mathbf{N}\begin{bmatrix}\mathbf{a}\\ \mathbf{b}\end{bmatrix} = p\begin{bmatrix}\mathbf{a}\\ \mathbf{b}\end{bmatrix}, \quad (S3.13)$$

where

$$\mathbf{N} = \begin{bmatrix}-\mathbf{T}^{-1}\mathbf{R}^T & \mathbf{T}^{-1}\\ \mathbf{R}\mathbf{T}^{-1}\mathbf{R}^T - \mathbf{Q} & -\mathbf{T}^{-1}\mathbf{R}^T\end{bmatrix}. \quad (S3.14)$$

The eigenvectors contain the polarization vectors $\mathbf{a}$ and traction vectors $\mathbf{b}$ of harmonic waves that satisfy the free surface boundary condition. The displacements and tractions for these solutions are

$$\mathbf{u} = \mathbf{a}e^{ik(\mathbf{m}\cdot\mathbf{x}+p\mathbf{n}\cdot\mathbf{x}-vt)}, \quad (S3.15)$$

$$\mathbf{t} = ik\mathbf{b}e^{ik(\mathbf{m}\cdot\mathbf{x}-vt)}. \quad (S3.16)$$

The Rayleigh wave is formed by a linear combination of wave modes whose amplitude must decay with depth. For this reason, we only consider solutions where p has a positive imaginary part. Below the limiting velocity of the material, the six eigenvalues occur in complex conjugate pairs, and so only three modes form the Rayleigh wave. Furthermore, the free surface condition implies that the linear combination of the tractions must be zero,

$$\sum_{i=1}^{3} c_i \mathbf{t}_i = \mathbf{0}. \qquad (S3.17)$$

This is often rewritten in the following form:

$$ik \begin{bmatrix} | & | & | \\ \mathbf{b}_1 & \mathbf{b}_2 & \mathbf{b}_3 \\ | & | & | \end{bmatrix} \begin{bmatrix} c_1 \\ c_2 \\ c_3 \end{bmatrix} e^{ik(\mathbf{m}\cdot\mathbf{x}-vt)} = \mathbf{Bc} = \mathbf{0}, \qquad (S3.18)$$

which has a nontrivial solution if and only if the determinant of $\mathbf{B}$ is zero.

To solve for the Rayleigh wave speed, one must employ an iterative algorithm. At each iteration, a trial wave speed v is considered, and the Stroh eigenvalue problem is solved to obtain the relevant eigenvectors, as described above. The traction vectors are extracted and used to form the matrix $\mathbf{B}$ and calculate its determinant. This process is repeated until the absolute value of the determinant is minimized. The resulting wave speed corresponds to the Rayleigh wave speed c_R.

Following this procedure for the same representative NITI material used in the previous section (ρ = 1000 kg/m^3, μ = 1 MPa, G = 20 kPa, and setting λ = 2.37 GPa so that the average longitudinal wave speed is approximately 1540 m/s), we found that the Rayleigh wave speed in a cornea-like NITI solid is approximately $\sqrt{G/\rho}$. The exact value varies slightly with the degree of anisotropy G/μ, from approximately 0.9553$\sqrt{G/\rho}$ in the isotropic limit ($G = \mu$) to $\sqrt{G/\rho}$ in the highly anisotropic limit ($G \ll \mu$) (Fig. S3.2).

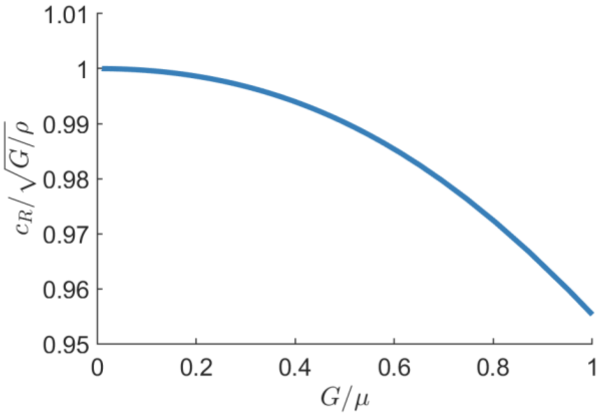

Supplementary Fig. S3.2. Rayleigh wave speed as a function of anisotropy. The Rayleigh wave speed is primarily governed by G in a NITI solid.

Supplementary Note 4. Analytical solution for guided wave modes in a NITI solid

The cornea can be modeled as an infinite layer of thickness h and density ρ bounded above by air and below by water (Fig. S4.1a). Assuming a NITI material model, the stiffness tensor (Fig. S4.1b) contains material constants

λ, μ, and G. Because acoustic micro-tapping generates a pseudo-line source, we also assume a plane strain state.

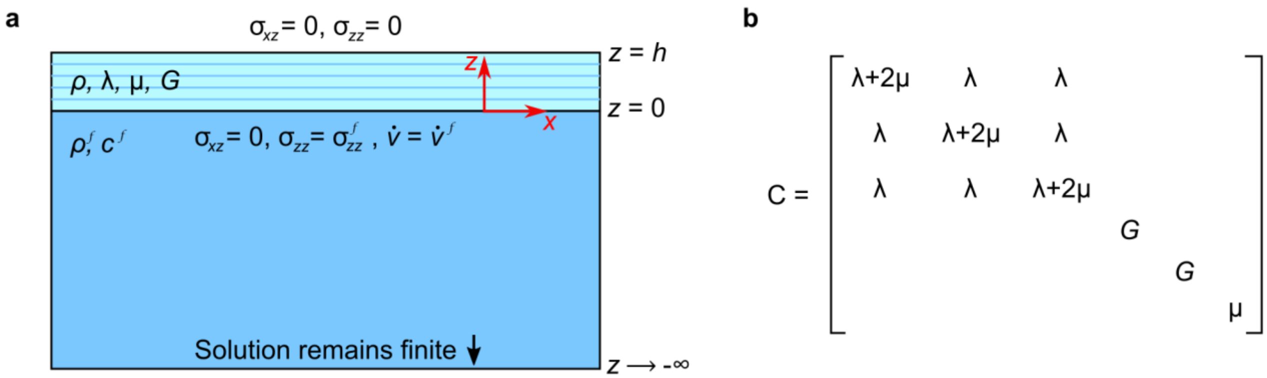

Supplementary Fig. S4.1. (a) Geometry and boundary conditions and (b) stiffness tensor for a NITI plate bounded above by air and below by water.

Introducing scales

Position: $\boldsymbol{x} \sim h$

Displacement: $\boldsymbol{u} \sim h$

Time: $t \sim h/\sqrt{\mu/\rho}$

Frequency: $f \sim \sqrt{\mu/\rho}/h$

Wavenumber: $k \sim h$

the dimensionless equations of motion yield the elastodynamic equations for a NITI material

$$u_{tt} = \beta^2 u_{xx} + \alpha^2 u_{zz} + \gamma^2 v_{xz} \quad (S4.1)$$

$$v_{tt} = \alpha^2 v_{xx} + \beta^2 v_{zz} + \gamma^2 u_{xz} \quad (S4.2)$$

$$\alpha^2 = \frac{G}{\mu} \quad (S4.3)$$

$$\beta^2 = \frac{\lambda + 2\mu}{\mu} \quad (S4.4)$$

$$\gamma^2 = \frac{\lambda + G}{\mu} \quad (S4.5)$$

Here, $\boldsymbol{u} = (u, v)$ is the dimensionless displacement field with x and z components, respectively; α, β, and γ are dimensionless parameters, and subscripts denote partial differentiation. We assume harmonic solutions for the displacements of the form

$$u(x, z, t) = Ae^{i(kx+\ell z-\omega t)} \quad (S4.6)$$

$$v(x, z, t) = Be^{i(kx+\ell z-\omega t)} \quad (S4.7)$$

where k and ℓ are dimensionless angular wavenumbers, ω is the dimensionless angular frequency, and A and B are arbitrary constants.

Substituting these solutions into the governing equations yields the homogeneous linear system

$$\begin{bmatrix} q_\beta^2 + \frac{\alpha^2}{\beta^2}\ell^2 & k\ell\frac{\gamma^2}{\beta^2} \\ k\ell\frac{\gamma^2}{\alpha^2} & q_\alpha^2 + \frac{\beta^2}{\alpha^2}\ell^2 \end{bmatrix}\begin{bmatrix} A \\ B \end{bmatrix} = \begin{bmatrix} 0 \\ 0 \end{bmatrix} \tag{S4.8}$$

where

$$q_\alpha^2 = k^2 - \frac{\omega^2}{\alpha^2} \tag{S4.9}$$

$$q_\beta^2 = k^2 - \frac{\omega^2}{\beta^2}. \tag{S4.10}$$

This has non-trivial solutions if and only if the determinant of the matrix is zero, resulting in a biquadratic equation for ℓ with four solutions

$$\ell^4 + \left[\frac{\alpha^2}{\beta^2}q_\alpha^2 + \frac{\beta^2}{\alpha^2}q_\beta^2 - \frac{\gamma^4 k^2}{\alpha^2\beta^2}\right]\ell^2 + q_\alpha^2 q_\beta^2 = 0 \tag{S4.11}$$

$$\ell = \pm\sqrt{\frac{1}{2}\left[\varphi \pm \sqrt{\varphi^2 - 4q_\alpha^2 q_\beta^2}\right]} \tag{S4.12}$$

$$\varphi = \frac{\gamma^4 k^2}{\alpha^2\beta^2} - \frac{\alpha^2}{\beta^2}q_\alpha^2 - \frac{\beta^2}{\alpha^2}q_\beta^2 \tag{S4.13}$$

Without loss of generality, we assume B = 1 and solve the corresponding coefficient A for each solution ℓ. This yields the following solutions, where the outer and inner ± signs match for each A and ℓ,

$$A = \pm\left[-\frac{\sqrt{2}\frac{\gamma^2 k}{\alpha^2}\sqrt{\varphi \pm \sqrt{\varphi^2 - 4q_\alpha^2 q_\beta^2}}}{\varphi + 2\frac{\beta^2}{\alpha^2}q_\beta^2 \pm \sqrt{\varphi^2 - 4q_\alpha^2 q_\beta^2}}\right] \tag{S4.14}$$

The full solutions are therefore linear combinations of four partial waves,

$$u(x,z,t) = \sum_{j=1}^{4} C_j A_j e^{i\ell_j z} e^{i(kx-\omega t)} \tag{S4.15}$$

$$v(x,z,t) = \sum_{j=1}^{4} C_j e^{i\ell_j z} e^{i(kx-\omega t)} \tag{S4.16}$$

In the fluid domain, we assume an acoustic material. The dimensionless acoustic wave equation in velocity potential form is

$$\dot{\boldsymbol{u}}^f = \nabla\Phi \tag{S4.17}$$

$$p^f = -\frac{\rho^f}{\rho}\Phi_t \qquad (S4.18)$$

$$\Delta\Phi - \frac{1}{\delta^2}\Phi_{tt} = 0 \qquad (S4.19)$$

$$\delta^2 = \frac{\rho\left(c^f\right)^2}{\mu} \qquad (S4.20)$$

The general solution for the fluid domain takes the form

$$\Phi = C_5 e^{\xi z} e^{i(kx-\omega t)} \qquad (S4.21)$$

$$\xi = \sqrt{k^2 - \frac{\omega^2}{\delta^2}} \qquad (S4.22)$$

$$\mathrm{Re}(\xi) > 0 \qquad (S4.23)$$

The constants C_j are chosen so that the solutions satisfy the non-dimensionalized boundary conditions:

$$\begin{array}{ll} \sigma_{xz} = 0 & \text{at } z = 1 \\ \sigma_{zz} = 0 & \text{at } z = 1 \\ \sigma_{xz} = 0 & \text{at } z = 0 \\ \sigma_{zz} = \sigma_{zz}^f & \text{at } z = 0 \\ \dot{v} = \dot{v}^f & \text{at } z = 0 \end{array} \qquad (S4.24)$$

where the superscripts f denote quantities in the fluid domain $z < 0$. Substituting the general solutions into the boundary conditions yields a 5×5 homogeneous system for the coefficients, $\mathbf{Mc} = \mathbf{0}$. This system has nontrivial solutions if and only if the determinant of the matrix $\mathbf{M}$ (equation S4.25) is zero. For a given frequency ω, wavenumbers k can be found that satisfy the dispersion relation using numerical root-finding methods or by minimizing the absolute value of the determinant

(S4.25)

$$\mathbf{M} = \begin{bmatrix} (\ell_1 A_1 + k)e^{i\ell_1} & (\ell_2 A_2 + k)e^{i\ell_2} & (\ell_3 A_3 + k)e^{i\ell_3} & (\ell_4 A_4 + k)e^{i\ell_4} & 0 \\ [k(\gamma^2 - \alpha^2)A_1 + \beta^2\ell_1]e^{i\ell_1} & [k(\gamma^2 - \alpha^2)A_2 + \beta^2\ell_2]e^{i\ell_2} & [k(\gamma^2 - \alpha^2)A_3 + \beta^2\ell_3]e^{i\ell_3} & [k(\gamma^2 - \alpha^2)A_4 + \beta^2\ell_4]e^{i\ell_4} & 0 \\ \ell_1 A_1 + k & \ell_2 A_2 + k & \ell_3 A_3 + k & \ell_4 A_4 + k & 0 \\ k(\gamma^2 - \alpha^2)A_1 + \beta^2\ell_1 & k(\gamma^2 - \alpha^2)A_2 + \beta^2\ell_2 & k(\gamma^2 - \alpha^2)A_3 + \beta^2\ell_3 & k(\gamma^2 - \alpha^2)A_4 + \beta^2\ell_4 & \omega\rho^f/\rho \\ \omega & \omega & \omega & \omega & -i\xi \end{bmatrix}$$

In Supplementary Note 2, we noted that our definition of a NITI material was isotropic in its longitudinal terms, but still can accurately approximate guided wave behavior in TI materials that are weakly anisotropic in their longitudinal terms.[29] The NITI dispersion relation S4.25 is a special case of the general dispersion relation for a TI solid, which may be solved in a similar manner as presented in this section. Using values for the stiffness coefficients from a recent Brillouin microscopy study[29] of the cornea, we take C_{11} = 2.971 GPa, C_{33} = 2.662 GPa, and $C_{13} + 2G$ = 2.680 GPa, along with what we consider to be representative values of G and μ for cornea (G = 20 kPa and μ = 1 MPa), and solve both the general and NITI dispersion relations. For all calculations, we assumed a layer thickness of 0.5 mm. We compare the A_0 mode for these two TI models and an isotropic model (G = μ = 20 kPa) to obtain the following:

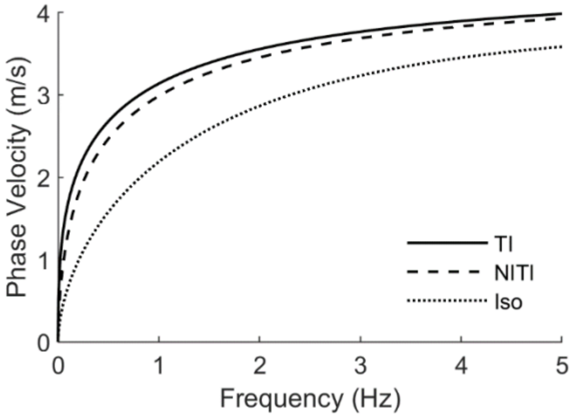

Supplementary Fig. S4.2. Comparison of the A_0 mode phase velocity spectra for a TI material with weak longitudinal anisotropy, NITI model, and an isotropic model. Both the NITI and TI models differ greatly from the isotropic model. Anisotropy in the longitudinal terms of the TI stiffness tensor is a second-order effect, and the NITI model captures most of the behavior, suggesting that the shear moduli μ and G dominate guided wave behavior.

The change from isotropic to a NITI model is rather large, while the change from NITI to a general TI model is very small. This shows that variations in C_{13} and C_{33} are second-order effects when examining guided shear waves in nearly incompressible TI materials. We argue that it is reasonable to neglect these terms, replacing them with the NITI model.

Supplementary Note 5. Finite element model of a NITI cornea

To provide an ideal, noise-free comparison to our experimental measurements, we developed a two-dimensional (plane strain) finite element model of guided wave propagation in a NITI material using OnScale (OnScale, Redwood City, CA). The model mirrors the configuration of our OCE experiments in porcine cornea. A thin elastic layer of thickness h = 0.55 mm and density ρ = 1000 kg/m^3 is bounded above by air (free surface condition) and below by a layer of water (Fig. S5a). The elastic layer is modeled as a NITI material with parameters λ, μ, and G, while the water layer is modeled as an isotropic

solid with a density ρ^f = 1000 kg/m³, shear wave speed $c_s = 0$, and longitudinal wave speed $c_L = \sqrt{(\lambda + 2\mu)/\rho}$.

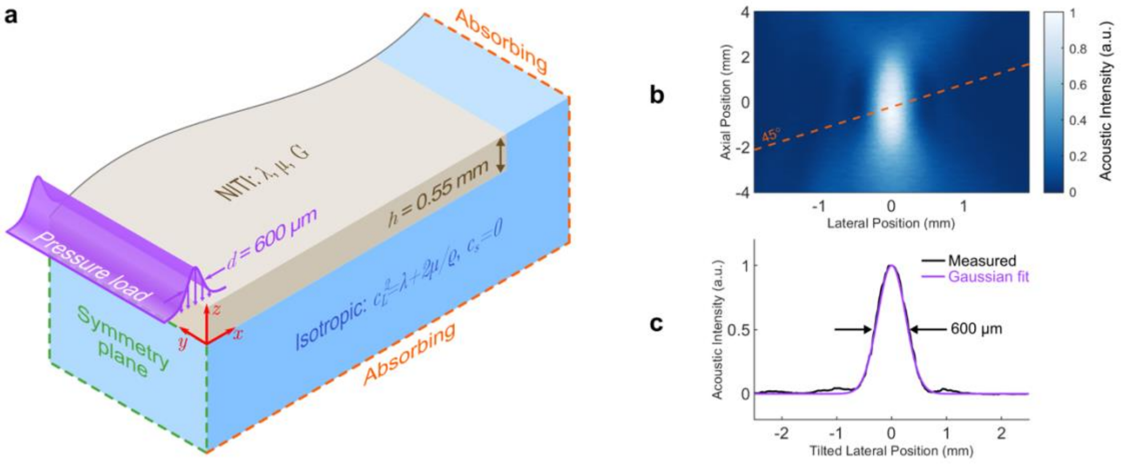

Supplementary Fig. S5. (a) Finite element model geometry used to simulate guided wave propagation in a NITI model of the cornea. (b) Acoustic intensity field measured from the air-coupled AμT transducer shows a nearly Gaussian profile (c) when sampled along a 45° line through the transducer focus.

The bottom and right boundaries of the domain function as absorbing conditions to minimize reflections as elastic waves leave the computational domain. To ensure stability at the boundary, the water layer extends along the right edge of the tissue domain and maintains the boundary condition. Symmetry is assumed with respect to the yz-plane. A temporally- and spatially-varying pressure load $P(x,t)$ is applied to the top boundary to mimic the AμT push of OCE experiments. The spatial profile is modeled as a Gaussian with a full-width-at-half-max (FWHM) d = 600 μm. This value was obtained by measuring the ultrasonic field of the AμT transducer with a needle hydrophone (HNC-1000, Onda, Sunnyvale, CA, USA) in air, sampling along a 45° line passing through the transducer focus (Fig. S5b and S5c). The temporal push profile is modeled by a super-Gaussian function with FWHM T = 100 μs. A small offset t_0 is included to avoid impulsive loading at t = 0. The full form of the pressure load is given by

$$P(x,t) = P_0 \exp\left[-4(\ln 2)\left(\frac{x}{d}\right)^2\right] \exp\left[-16(\ln 2)\left(\frac{t-t_0}{T}\right)^4\right], \qquad (S5.1)$$

where the pressure amplitude P_0 is an arbitrary constant (5 kPa in this study).

Biological tissue is nearly incompressible, exhibiting longitudinal wave speeds on the order of 1540 m/s and shear wave speeds in the range of 1-10 m/s. This corresponds to a Poisson's ratio of $\nu \approx 0.4999995$. In practice, it is not necessary to directly model this exact value of Poisson's ratio, and solutions for $\nu >$ 0.4995 converge to the incompressible solution.[35] In this study, we enforce this constraint for all models by requiring that the ratio $\sqrt{(\lambda + 2\mu)/\mu}$ = 35. In the isotropic limit $G = \mu$, this gives a Poisson's ratio of ν = 0.4996.

The computational domain was discretized using linear finite elements on a regular rectangular grid with a minimum of 40 elements per elastic wavelength, as approximated by the push width. Belytchko-Bindeman hourglass suppression was applied to prevent spurious modes from corrupting the solution. All simulations used an explicit time-stepping method to generate the full displacement and velocity

fields at each space and time point. However, only the vertical velocity component was used in our analysis. This is analogous to OCE experiments where only this component is available.

Supplementary Note 6. Finite element model to investigate the effect of corneal curvature

In the analytical solution described in Supplementary Note 4 and the finite element model described in Supplementary Note 5, we model the cornea as a flat NITI layer bounded above by air and below by water. In reality, the cornea is not flat, but rather curved, and this curvature could affect guided wave behavior. In particular, the dispersion relation of guided waves in a curved plate varies from the flat plate case based on the relationship between the wavelength, the radius of curvature, and the layer thickness. Krauklis and Molotkov [44] derived dispersion relations for azimuthal modes in a cylindrical shell and meridian modes in a spherical shell and classified dispersion behavior in two regimes:

$$(1)\ \frac{\lambda}{R} > \frac{h}{\lambda} \qquad (S6.1)$$

$$(2)\ \frac{\lambda}{R} < \frac{h}{\lambda}$$

When condition (1) holds, dispersion is strongly influenced by the curvature of the shell, and dispersion is stronger for spherical modes than cylindrical modes. Condition (2) corresponds to mild curvature, and the dispersion is mainly governed by the terms obtained for a flat plate.

The expected elastic wavelength is determined by some combination of the product $c_s T$ and d, where c_s is the bulk shear wave speed of the medium, T is the excitation duration, and d is the excitation width. Generally, when the ratio $c_s T/d$ is small, pressure confinement is achieved and the expected wavelength is on the order of d. In contrast, when the ratio is large, the wavelength is on the order of $c_s T$. For a typical acoustic micro-tapping excitation, we have T = 100 µs and d = 500 µm. For c_s = 4 m/s, this gives $c_s T/d$ = 0.8, and the wavelength λ is on the order of 0.4-0.5 mm. Combining these estimates (and assuming R = 6.5 mm, h = 0.5 mm), we obtain $\lambda/R \approx$ 0.08 and $h/\lambda \approx$ 1. Thus, we expect condition (2) to hold, and curvature can be neglected.

For additional validation, we developed a finite element model in OnScale to estimate the effect of curvature on the dispersion behavior. This model was similar to that described in Supplementary Note 5, but featured a few distinct differences in both geometry and post-processing.

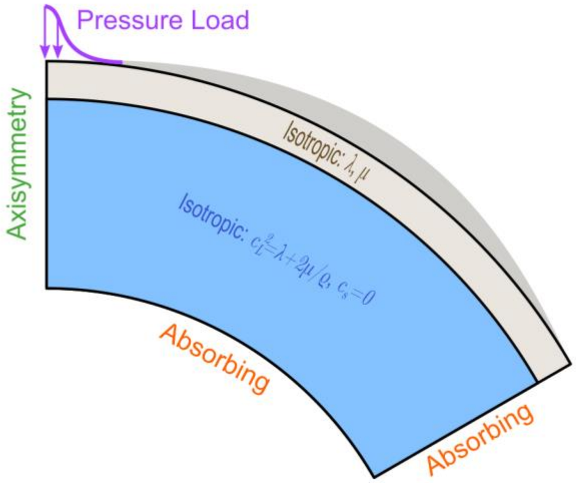

Supplementary Fig. S6.1. Curved geometry used to study guided waves in a spherically curved layer. The full model geometry can be considered a solid of revolution obtained by revolving the domain shown about the axisymmetric boundary.

The finite element model consisted of a rectangular domain of thickness h = 0.55 mm bent to an outer radius of curvature of 6.5 mm (Supplementary Fig. S6.1). Here we assume an axisymmetric rather than a plane strain state. The tissue layer was assumed to be isotropic with density ρ = 1000 kg/m^3, shear wave speed c_S = 4 m/s, and longitudinal wave speed c_L = 35c_S. The tissue layer sat atop a simulated water layer, modeled as an elastic solid with shear wave speed c_S = 0 m/s and a density and longitudinal wave speed matched to the tissue layer. Outer edges of the domain were set as absorbing boundaries. The spatio-temporal push profile was defined as in Supplemental Note 5, with a Gaussian profile in space and a super-Gaussian profile in time. The domain was discretized with linear finite elements on a regular conformal grid with at least 40 elements per elastic wavelength.

Solving this model produces a pseudo-point source solution, a numerical approximation to the Green's function for this problem. The wave field solution for a line source, such as the one used in our AµT experiments, can be obtained by convolving the Green's function with a source distribution. We numerically evaluated this convolution integral along a linear path on the spherical surface using trapezoid integration. This yielded the wave field along the entire spherical surface. Supplemental Video 1 shows the results. In general, the wave field is more complex compared to the flat plate plane strain approximation. However, extracting the wave field from the mid-plane normal to the line source (as would be measured using OCE), we find that the flat plate and spherical models produce very similar wave fields (Supplementary Fig. S6.2, a-b). Analyzing the 2D Fourier spectrum of the spherical model along this mid-plane, we find that the isotropic dispersion relation for a flat plate[42] provides a close fit to the numerical solution (Supplementary Fig. S6.2, c). This is expected, as the radius of curvature of the cornea is large relative to its thickness.[44,45] Thus, it appears reasonable to neglect corneal curvature in analyzing guided wave behavior in the cornea. While these computations were performed using an isotropic model, we expect a NITI model to produce similar results.

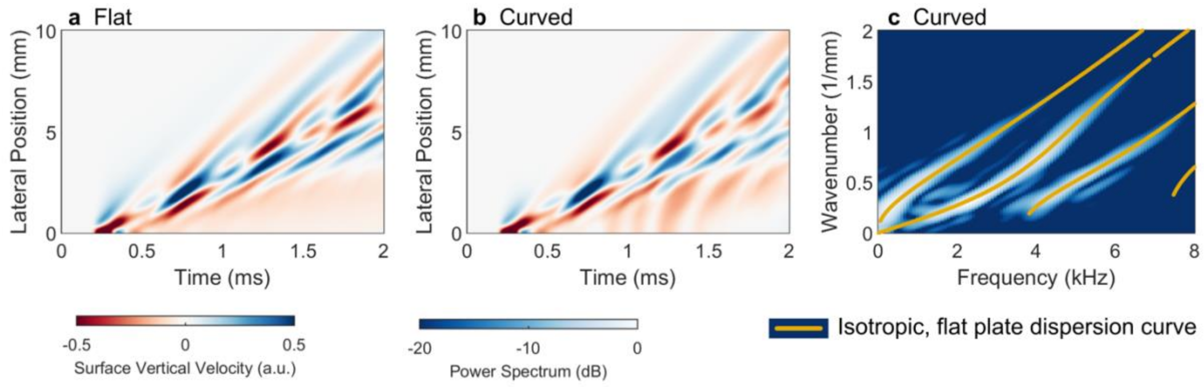

Supplementary Fig. S6.2. Surface vertical velocity wave fields for a simulated (a) flat plate and (b) spherically curved layer are very similar. (c) The 2D Fourier spectrum for the spherically curved layer closely matches the theoretical dispersion curves obtained for a flat plate (yellow markers).

Supplementary Note 7. Fitting experimental data with the NITI model

Quantitative moduli estimates in bounded materials require a method to determine the dispersion relation most closely matching observed guided wave modes. In a typical dynamic OCE experiment, the OCT signal phase difference is used to reconstruct the vertical component of the velocity field. We extract the surface part of this signal to create an XT representation of the wave field and apply a two-dimensional Fourier transform to produce a frequency-wavenumber (FK) spectrum. This spectrum can then be compared to a theoretical dispersion relation to obtain modulus estimates.

Our study differs from many dynamic OCE studies in how we analyze the FK spectrum. A common approach is to algorithmically identify the local peaks of the FK spectrum. They ideally trace the central portions of the modes and can, therefore, be used to produce "measured" dispersion curves to compare against theoretical solutions. Typically, these peaks are first converted to the frequency-phase velocity (FC) domain using the relationship between the phase velocity c (in m/s), frequency f (in Hz), and wavenumber k (in m^{-1}): $c = f/k$.

In Figure S7.1, we reproduce FK maps from Figure 4 for PVA phantoms and porcine cornea to illustrate that this process is not without error. Applying a peak-finding algorithm to the FK spectra (Figures S7.1a, S7.1d) yields the narrow dispersion curves shown in Figures S7.1b and S7.1e. They are then converted to phase velocities (Figures S7.1c and S7.1f). The peak-finding algorithm requires tuning various parameters such as minimum peak heights, minimum peak distances, and minimum peak prominence. Even with well-chosen parameters, spurious points may be chosen that are not related to the mode (for example, Figures S7.1e-f). Compared to the full FK spectra, this representation ignores the fact that these points contain much less energy than the points covered by the best-fit dispersion curves. These points must either be manually cleaned from the data prior to fitting dispersion curves or weighted carefully to ensure that they do not introduce fit errors (Note that the fits shown in Figure S7.1 are based on our FK-domain method and do not use spectral peaks). Another technical challenge with spectral peaks is that more than one peak may be returned for each frequency. This follows physically from the dispersion relation, where multiple modes can exist at any given frequency. However, spurious peaks make it difficult to algorithmically sort modes from noise.

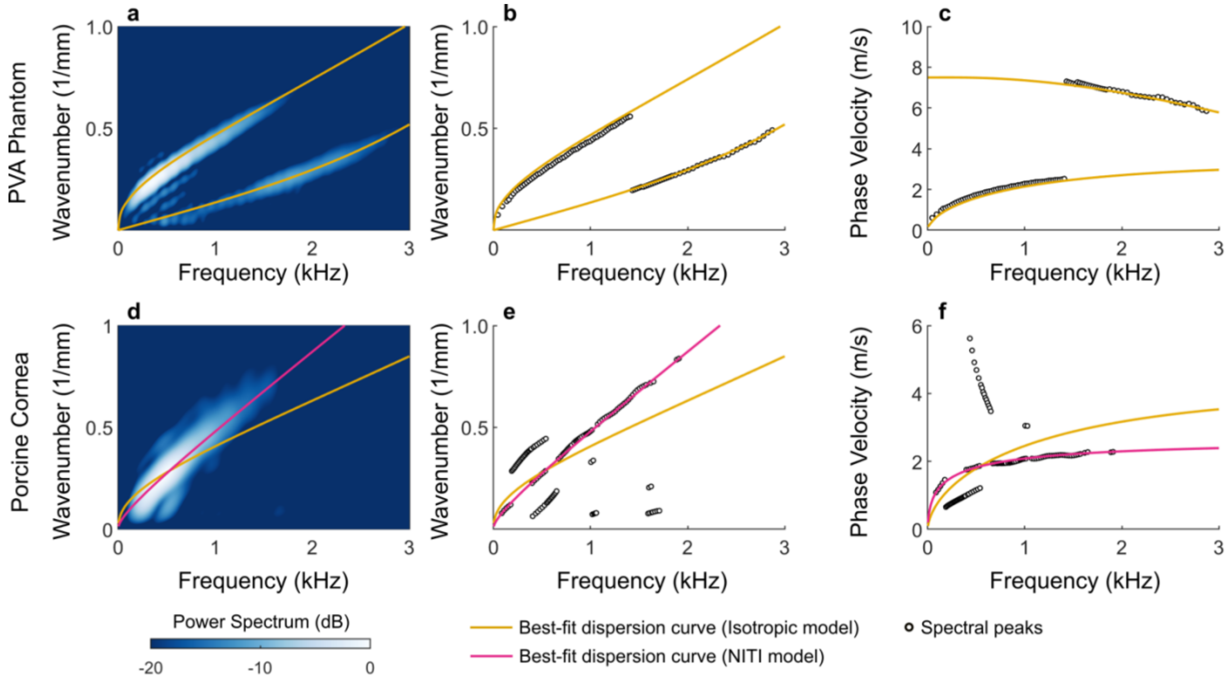

Supplementary Figure S7.1. Typical method of dynamic OCE dispersion analysis shown for PVA phantom (a-c) and porcine cornea (d-f) data, presented in Figure 4. (a,d) The FK spectra are computed from the surface XT fields, shown here over a 20 dB dynamic range. (b,e) The local peaks of the FK spectra are identified algorithmically (white circles). (c,f) These peaks are then converted to the frequency-phase velocity representation and compared with theoretical solutions to the dispersion relation (yellow and pink lines).

To avoid these issues, we developed a fitting routine based on the full FK spectra. It finds the dispersion curve that overlaps with the maximum amount of spectral power. Supplementary equation S4.25 acted as the forward model for the dispersion curves. A number of physical parameters were considered fixed, including the corneal density (ρ = 1000 kg/m^3), corneal longitudinal wave speed (c_L = 1540 m/s), and mean corneal thickness (measured from B-mode OCT images). The cornea was bounded from below by water with a density of 1000 kg/m^3 and longitudinal wave speed of 1480 m/s. Because we did not observe the S_0 mode in corneal measurements, we extracted only the A_0 mode from the forward model using a mode-tracing routine (similar to Pavlakovic et. al [46]).

Fitting the theoretical dispersion relation was performed by maximizing the following objective function:

$$\Phi(\mu, G) = \frac{1}{N_f} \sum_f \sum_k w(f, k; \mu, G) |\hat{v}(f, k)|^2 - \beta \left| \frac{\mu}{\lambda} \right| \qquad (S7.1)$$

Here, $\hat{v}$ is the normalized 2D Fourier spectrum of the measured surface velocity data. The function $w(f, k; \mu, G)$ is related to the A_0 mode solution for a NITI or isotropic ($G = \mu$) material. The first term describes the energy covered by the dispersion curve, normalized by the number of FFT bins included in the fit (N_f). The second acts as a regularization term that ensures that the ratio μ/λ remains small, thus satisfying the nearly-incompressible assumption. In equation S7.1, β was set to 1, based on an L-curve analysis.[47] The value of λ was updated at each iteration according to $\lambda = \rho c_L^2 - 2\mu$. At a given frequency f and given the current parameter set (μ, G), the dispersion relation solver returns the wavenumber

associated with the A_0 mode, k_0. Let Δk be the sampling interval in the wavenumber. The weight function w is then defined as

$$w(f,k;\mu,G) = \begin{cases} e^{-\frac{1}{2}\left(\frac{k-k_0(f,\mu,G)}{1.2\Delta k}\right)^2}, & |k-k_0(f,\mu,G)| \le 3\Delta k \\ 0, & \text{otherwise} \end{cases} \tag{S7.2}$$

That is, the weights are assigned at each frequency with a peak value at the wavenumber of the theoretical dispersion curve and weights decaying (with a Gaussian distribution) as the distance from the theoretical curve increases.

Note that this optimization is not posed as a least-squares error problem. Rather, we seek to maximize the spectral energy contained in a small weighted window around the A_0 mode. The dispersion relation estimates the location of the A_0 mode (e.g. the wavenumber of the mode for a given frequency), but cannot predict the spectral energy in a frequency-wavenumber bin (which depends on the method used to excite guided waves).

To compare the isotropic and NITI fits in this study, we defined a goodness-of-fit metric based on an "unconstrained, global optimum" for the objective function, $\Phi_{\max}$. At each frequency f, we solve the following optimization problem:

$$k_{\max} = \underset{\tilde{k}}{\operatorname{argmax}} \sum_k \tilde{w}(f,k;\tilde{k},\mu,G)|\hat{v}(f,k)|^2 \tag{S7.3}$$

$$\tilde{w}(f,k;\tilde{k},\mu,G) = \begin{cases} e^{-\frac{1}{2}\left(\frac{k-\tilde{k}}{1.2\Delta k}\right)^2}, & |k-\tilde{k}| \le 3\Delta k \\ 0, & \text{otherwise} \end{cases} \tag{S7.4}$$

$$\Phi_{\max}(\mu,G) = \frac{1}{N_f}\sum_f \sum_k \tilde{w}(f,k;k_{\max},\mu,G)|\hat{v}(f,k)|^2 \tag{S7.5}$$

This is equivalent to finding the Gaussian-weighted window containing the most spectral energy at each frequency independently and summing those contributions to the total mode energy. Since $k_{\max}$ may vary at each frequency independently from the dispersion relation, $k_{\max}$ may not follow the mode shape exactly and may not even be smooth. However, this means that $\Phi_{\max}$ represents an upper bound on the value of Φ calculated during fitting. We therefore compare the following goodness-of-fit measures:

$$g_{\text{iso}} = \frac{\Phi_{\text{iso}}}{\Phi_{\max}} \tag{S7.6}$$

$$g_{\text{NITI}} = \frac{\Phi_{\text{NITI}}}{\Phi_{\max}} \tag{S7.7}$$

Thus, g_{NITI} and g_{iso} indicate what portion of the maximum possible mode energy is captured by a given A_0 dispersion curve for NITI and isotropic models, respectively, with values near 1 indicating that the theoretical dispersion curve captures almost all of the mode's energy.

References

1. Anderson, K., El-Sheikh, A. & Newson, T. Application of structural analysis to the mechanical behaviour of the cornea. *J. R. Soc. Interface* **1**, 3–15 (2004).

2. Bekesi, N., De La Hoz, A., Kling, S. & Marcos, S. The Effects of Cross-linking on the Static and Dynamic Corneal Viscoelastic Properties. *Assoc. Res. Vis. Ophthalmol.* 520461 (2014).

3. Bekesi, N., Dorronsoro, C., De La Hoz, A. & Marcos, S. Material properties from air puff corneal deformation by numerical simulations on model corneas. *PLoS One* **11**, (2016).

4. Boschetti, F., Triacca, V., Spinelli, L. & Pandolfi, A. Mechanical characterization of porcine corneas. *J. Biomech. Eng.* **134**, (2012).

5. Elsheikh, A., Alhasso, D. & Rama, P. Biomechanical properties of human and porcine corneas. *Exp. Eye Res.* **86**, 783–790 (2008).

6. Elsheikh, A. & Alhasso, D. Mechanical anisotropy of porcine cornea and correlation with stromal microstructure. *Exp. Eye Res.* **88**, 1084–1091 (2009).

7. Han, Z. *et al.* Quantitative assessment of corneal viscoelasticity using optical coherence elastography and a modified Rayleigh – Lamb equation of corneal viscoelasticity equation. *J. Biomed. Opt.* **20**, 2–5 (2015).

8. Han, Z. *et al.* Optical coherence elastography assessment of corneal viscoelasticity with a modified Rayleigh-Lamb wave model. *J. Mech. Behav. Biomed. Mater.* **66**, 87–94 (2017).

9. Hatami-Marbini, H. & Etebu, E. An experimental and theoretical analysis of unconfined compression of corneal stroma. *J. Biomech.* **46**, 1752–1758 (2013).

10. Hatami-Marbini, H. & Etebu, E. Hydration dependent biomechanical properties of the corneal stroma. *Exp. Eye Res.* **116**, 47–54 (2013).

11. Hatami-Marbini, H. Viscoelastic shear properties of the corneal stroma. *J. Biomech.* **47**, 723–728 (2014).

12. Kling, S., Ginis, H. & Marcos, S. Corneal biomechanical properties from two-dimensional corneal flap extensiometry: Application to UV-Riboflavin cross-linking. *Investig. Ophthalmol. Vis. Sci.* **53**, 5010–5015 (2012).

13. Kling, S., Bekesi, N., Dorronsoro, C., Pascual, D. & Marcos, S. Corneal viscoelastic properties from finite-element analysis of in vivo air-puff deformation. *PLoS One* **9**, (2014).

14. Singh, M. *et al.* Noncontact Elastic Wave Imaging Optical Coherence Elastography for Evaluating Changes in Corneal Elasticity Due to Crosslinking. *IEEE J. Sel. Top. Quantum Electron.* **22**, 1–32 (2017).

15. Wollensak, G., Spoerl, E. & Seiler, T. Stress-strain measurements of human and porcine corneas after riboflavin-ultraviolet-A-induced cross-linking. *J. Cataract Refract. Surg.* **29**, 1780–1785 (2003).

16. Zeng, Y., Yang, J., Huang, K., Lee, Z. & Lee, X. A comparison of biomechanical properties between human and porcine cornea. *J. Biomech.* **34**, 533–537 (2001).

17. Hoeltzel, D., Altman, P., Buzard, K. & Choe, K. Strip extensiometry for comparison of the

mechanical response of bovine, rabbit, and human corneas. *J. Biomech. Eng.* **114**, 202-215. (1992).

18. Kling, S. *et al.* Numerical model of optical coherence tomographic vibrography imaging to estimate corneal biomechanical properties. *J. R. Soc. Interface* **11**, 20140920–20140920 (2014).

19. Petsche, S. J., Chernyak, D., Martiz, J., Levenston, M. E. & Pinsky, P. M. Depth-Dependent Transverse Shear Properties of the Human Corneal Stroma. **53**, (2017).

20. Sloan, S. R., Khalifa, Y. M. & Buckley, M. R. The location- and depth-dependent mechanical response of the human cornea under shear loading. *Investig. Ophthalmol. Vis. Sci.* **55**, 7919–7924 (2014).

21. Bryant, M., Szerenyi, K., Schmotzer, H. & McFonnell, P. Corneal tensile strength in fully healed radial keratotomy wounds. in *Invest Ophthalmol Vis Sc* 35(7):3022–31 (1994).

22. Elsheikh, A., Wang, D. & Pye, D. Determination of the modulus of elasticity of the human cornea. *J. Refract. Surg.* **23**, 808–818 (2007).

23. Litwiller, D. V. *et al.* MR elastography of the ex vivo bovine globe. *J. Magn. Reson. Imaging* **32**, 44–51 (2010).

24. Bekesi, N., Kochevar, I. E. & Marcos, S. Corneal biomechanical response following collagen cross-linking with Rose Bengal-green light and riboflavin-UVA. *Investig. Ophthalmol. Vis. Sci.* **57**, 992–1001 (2016).

25. Singh, M. *et al.* Quantifying the effects of hydration on corneal stiffness with noncontact optical coherence elastography. *J. Cataract Refract. Surg.* **44**, 1023–1031 (2018).

26. Petsche, S. J., Chernyak, D., Martiz, J., Levenston, M. E. & Pinsky, P. M. Depth-dependent transverse shear properties of the human corneal stroma. *Investig. Ophthalmol. Vis. Sci.* **53**, 873–880 (2012).

27. Singh, M. *et al.* Assessing the effects of riboflavin/UV-A crosslinking on porcine corneal mechanical anisotropy with optical coherence elastography. *Biomed. Opt. Express* **8**, 349–366 (2017).

28. O'Donnell, M. & Skovoroda, A. R. Prospects for elasticity reconstruction in the heart. *IEEE Trans. Ultrason. Ferroelectr. Freq. Control* **51**, 322–328 (2004).

29. Eltony, A. M., Shao, P., & Yun, S.-H. Measuring mechanical anisotropy of the cornea with Brillouin microscopy. arXiv:2003.04344v1 (2020)

30. Ambroziński, Ł. *et al.* Acoustic micro-tapping for non-contact 4D imaging of tissue elasticity. *Sci. Rep.* **6**, 38967 (2016).

31. Nguyen, T. D. & Boyce, B. L. An inverse finite element method for determining the anisotropic properties of the cornea. *Biomech. Model. Mechanobiol.* **10**, 323–337 (2011).

32. Li, J. *et al.* Revealing anisotropic properties of cornea at different intraocular pressures using optical coherence elastography. *Opt. Elastography Tissue Biomech. III* **9710**, 97100T (2016).

33. Singh, M. *et al.* Investigating Elastic Anisotropy of the Porcine Cornea as a Function of Intraocular Pressure With Optical Coherence Elastography. *J. Refract. Surg.* **32**, 562–567 (2016).

34. Timoshenko, S. & Woinowsky-Krieger, S. *Theory of plates and shells*. 2nd ed. McGraw-Hill, New York. (1959).

35. Pelivanov, I. *et al.* Does group velocity always reflect elastic modulus in shear wave elastography? *J. Biomed. Opt.* **24**, 1 (2019).

36. Barnett, D. M. Synthesis of the sextic and the integral formalism for dislocations, Green's function and surface waves in anisotropic elastic solids." *Phys. Norv* **13**, (1973).

37. Chadwick, P. & Smith, G. Foundations of the Theory of Surface Waves in Anisotropic Elastic Materials. In: *Advances in Applied Mechanics* **17**, 303–376 (1977).

38. Ting, T. C. T. The Stroh Formalism. In: *Anisotropic Elasticity, Theory and Applications.* 155–184 (1996).

39. Tanuma, K. Stroh Formalism and Rayleigh Waves. *J Elasticity* **89**, 5–154 (2007).

40. Cherry, M. R., Sathish, S. & Grandhi, R. A numerical method for predicting Rayleigh surface wave velocity in anisotropic crystals. *J. Comput. Phys.* **351**, 108–120 (2017).

41. Payton, R. G. Elastic wave propagation in transversely isotropic media. Martinus Nijhoff Publishers, The Hague, Netherlands (1983).

42. Kirby, M. A. *et al.* Optical coherence elastography in ophthalmology. *J. Biomed. Opt.* **22**, 1 (2017).

43. Auld, B. Acoustic fields and waves in solids. R.E. Krieger., Malabar, Florida (1990).

44. Krauklis, P. V. & Molotkov, L. A. Low-frequency lamb waves in cylindrical and spherical layers in an elastic medium. *J. Soviet Mathematics* **3**, 82–90, (1975).

45. Brekhovskikh, L. M. *Waves in Layered Media*. Academic Press, New York (1976).

46. Pavlakovic, B., Lowe, M., Alleyne, D. & Cawley, P. Disperse: A General Purpose Program for Creating Dispersion Curves. In: *Review of Progress in Quantitative Nondestructive Evaluation*. 185–192 (1997).

47. Hansen, P. C. Analysis of discrete ill-posed problems by means of the L-curve. SIAM Review, 34(4), 561–580 (1992).
