# Supplementary material for: Nearly-incompressible transverse isotropy (NITI) of cornea elasticity: model and experiments with acoustic micro-tapping OCE
Source: Sci Rep. 2020 Jul 31;10:12983. doi: 10.1038/s41598-020-69909-9 (PMC7395720; doi:10.1038/s41598-020-69909-9)
Supplement: Supplementary file 2 — Supplementary Information 2. [file 41598_2020_69909_MOESM2_ESM.pdf]

Supplementary Software Documentation

Nearly-incompressible transverse isotropy (NITI) of cornea elasticity: model and experiments with acoustic micro-tapping OCE

John J. Pitre Jr.[1*], Mitchell A. Kirby[1*], David S. Li[1,2], Tueng T. Shen[3], Ruikang K. Wang[1,3], Matthew O'Donnell[1], and Ivan Pelivanov[1]

[1.] University of Washington, Department of Bioengineering, Seattle, Washington, United States
[2.] University of Washington, Department of Chemical Engineering, Seattle, Washington, United States
[3.] University of Washington, Department of Ophthalmology, Seattle, Washington, United States

Supplementary Software Documentation

To aid and accelerate future studies in translating corneal elasticity measurements towards clinical use, we include a Supplementary Software Library that will allow other investigators to apply our findings to their own work. This includes

(i) MATLAB functions to solve the analytical guided wave model (eq. S4.25 in Supplementary Note 4);

(ii) Finite element model (FEM)-based OnScale script to obtain a 2D field of propagating mechanical waves in the NITI layer corneal model;

(iii) Inversion routine for recovering both moduli G and μ from measured OCE wavefields;

(iv) OnScale script to simulate guided wave propagation in a spherical layer.

Below, we provide basic instructions to run the included scripts and functions. Each file is extensively commented to explain its use and implementation. In addition, we provide some example data files that can be used to reproduce some of the analyses included in the main paper and supplementary notes.

Example Data

To facilitate the examples, we include three example data files in *.mat format (MATLAB, MathWorks, Natick, MA):

- *example_porcine_cornea_data.mat* : Contains an example of experimental OCE measurements of guided wave propagation in ex vivo porcine cornea (IOP = 10 mmHg). The included data arrays are: (v) the vertical velocity component (in arbitrary units) at the corneal surface, (x) the spatial positions, (t) the time points, (h) the cornea thickness, (dx) the spatial sampling, and (dt) the temporal sampling. Note that this example may require up to 9 GB of RAM.
- *example_onscale_results_niti_guided.mat* : A data structure containing pre-computed results from the OnScale finite element model *niti_guided.flxinp*.
- *example_onscale_results_point_source_curved.mat* : A data structure containing pre-computed results from the OnScale finite element model *point_source_curved.flxinp*.

Example Scripts

We provide three example scripts that reproduce similar analyses as those found in the main paper and supplementary notes. These can be run from the MATLAB command prompt or editor.

- *example_compute_dispersion_curves.m* : Computes A_0 dispersion curves for a nearly-incompressible transversely isotropic (NITI) layer bounded above by air and below by water for varying degrees of anisotropy. This demonstrates how to use the included functions to solve the analytical dispersion relation (Supplementary Equation S4.25). The output resembles Figures 2a and 2c.
- *example_load_onscale_niti_results.m* : Loads OnScale results from the NITI guided wave model (*niti_guided.flxinp*) and generates an XT plot and 2D Fourier power spectrum plot. The output resembles Figures 3c and 3g.
- *example_run_niti_fit.m* : Runs the inversion routine to determine the best fit elastic moduli for a NITI (G and μ) and isotropic (only μ) layer. The script loads the example experimental OCE data from ex vivo porcine cornea (IOP = 10 mmHg), computes the 2D Fourier power spectrum, and runs the fitting routines. The output resembles Figure 5b.
- *example_run_onscale_spherical_layer_AuT.m* : Runs the analysis presented in Supplementary Note 6 for guided wave propagation in a spherically curved layer due to a line source incident on the surface. The outputs resemble Supplementary Video 1 and Supplementary Figure S6.2.

List of Functions and Scripts

The following MATLAB functions and OnScale scripts are included in the Supplementary Software. Each is extensively documented with top-level comments that describe input parameters, outputs, and notes. Additional implementation details are included throughout. Here, we include brief descriptions of each. Functions are grouped based on their use.

Analytical guided wave model of the cornea

- *compute_niti_amode.m* : Computes the phase velocity spectrum for the A_0 mode of a NITI layer of thickness h bounded above by air and below by water.
- *compute_niti_kappa.m* : Computes the condition number and determinant of the characteristic matrix M (defined by Supplementary Equation S4.25) for a NITI layer over a range of requested frequencies and wavenumbers.
- *compute_niti_characteristic_matrix.m* : Computes the characteristic matrix M (defined by Supplementary Equation S4.25) for a NITI layer.

Finite element model (OnScale) of guided wave propagation in a NITI layer corneal model

- *niti_guided.flxinp* : OnScale input file that can be run in OnScale analyst mode to simulate guided wave propagation in the cornea assuming a NITI model. The file can also be opened in any text editor to examine the model. The output generated by the OnScale solver is a series of binary *.flxdato files, each containing the surface vertical velocity field at a given time step. The full set of results can be read into MATLAB using the helper function *load_onscale_flexdata.m*, described below.

Inversion routine for recovering moduli G and μ from measured OCE wavefields

- *fit_spectrum_niti.m* : Compute the best-fit moduli for an input 2D Fourier power spectrum, assuming a NITI model (using only the A_0 mode). Returns both moduli G and μ.
- *fit_spectrum_iso.m* : Compute the best-fit moduli for an input 2D Fourier power spectrum, assuming an isotropic model (using only the A_0 mode). Returns a single isotropic modulus μ.
- *xttools_power_spectrum.m* : Computes the 2D Fourier power spectrum from the input XT surface vertical velocity field.

Guided wave propagation in a spherical layer

- *point_source_curved.flxinp* : OnScale input file that can be run in OnScale analyst mode to simulate guided wave propagation in a spherically curved layer. The guided waves are assumed to be axisymmetric and excited from a point-like source at the apex of the sphere. These results are then used to approximate the line-source solution. The output generated by the OnScale solver is a series of binary *.flxdato files, each containing the surface vertical velocity field at a given time step. The full set of results can be read into MATLAB using the helper function *load_onscale_flexdata.m*, described below.
- *onscale_point_source_curved_integral.m* : MATLAB function that convolves the point-source solution on a spherical layer with a source distribution to simulate guided wave excitation by a line source on a spherical layer.

Additional helper files for processing OnScale results

- *load_onscale_matdata.m* : Loads a set of OnScale result output *.mat files from a directory into a MATLAB structure.
- *load_onscale_flexdata.m* : Loads a set of OnScale result output *.flxdato files from a directory into a MATLAB structure.
- *read_flxdato.m* : helper function for reading the contents of *.flxdato binary files into MATLAB

System/Software Requirements

All computations carried out in this study were completed on a Windows 10 based workstation with an Intel Core i7-4790 CPU (3.6 GHz) with 16 GB RAM. OnScale models were run in the cloud from OnScale Analyst (v 1.29.1.0) using double precision arithmetic. MATLAB release R2018a was used for all processing. We recommend using a computer with at least 8 GB of RAM to run all example scripts.
